# Lagged influence of Atlantic and Pacific climate patterns on European extreme precipitation

**DOI:** 10.1038/s41598-018-24069-9

**Published:** 2018-04-10

**Authors:** Hossein Tabari, Patrick Willems

**Affiliations:** 1 KU Leuven, Department of Civil Engineering, Hydraulics Division, Leuven, Belgium; 2 Vrije Universiteit Brussel, Department of Hydrology and Hydraulic Engineering, Brussels, Belgium

## Abstract

The risk of European extreme precipitation and flooding as an economic and humanitarian disaster is modulated by large-scale atmospheric processes that operate over (multi-)decadal periods and transport huge quantities of moisture inland from the oceans. Yet the previous studies for better understanding of extreme precipitation variability and its skillful seasonal prediction are far from comprehensive. Here we show that the winter North Atlantic Oscillation (NAO) and, to a lesser extent, winter ENSO signal have a controlling influence not only concurrently on European extreme precipitation anomaly in winter, but in a delayed way on the extremes in the following seasons. In a similar pattern, there is a strong footprint of summer atmospheric circulations over the Mediterranean Sea on summer extreme precipitation and with 1-, 2- and 3-season lags on the following autumn, winter and spring extremes. The combined influences of the different atmospheric circulation patterns mark a significant step forward for an improved predictability of European extreme precipitation in the state-of-the-art seasonal prediction systems.

## Introduction

Extreme weather events such as extreme precipitation and flooding have become more frequent and intense over time^1–6^. Recent extreme events in Europe such as devastating floods in central Europe in August 2002 (losses: US$ 16,500 million; fatalities: 39) and May/June 2013 (losses: US$ 12,600 million; fatalities: 25) and in England and Wales in June/July 2007 (losses: US$ 8,000 million; fatalities: 5) resulted in severe damages and fatalities^7^.

This has prompted a concern to characterize the processes leading to the extreme events jeopardizing human life. The first base to better comprehension of the responsible mechanisms is to pinpoint the source of moisture for such a huge event, by keeping in mind the fact that precipitation in any place in the world has its origin in water evaporating from large water bodies such as oceans. The major sources of land precipitation over Europe have recently been identified. They differ from a season to another, with the North Atlantic providing most of the moisture for winter precipitation and the Mediterranean Sea for summer precipitation^8–10^. With these known sources of moisture, a question arises as to how much the variability of heavy precipitation resulting in destructive floods can be explained by atmospheric circulation patterns defined over these moisture sources. The North Atlantic Oscillation (NAO) index expressing the variability in the strength of the meridional dipole anomaly in sea level pressure (SLP) over the North Atlantic moisture source has been recognized as the leading mode of the European climate variability during the boreal winter^11–15^. Whilst the main atmospheric driver of winter precipitation variability is well established, that of summer precipitation remains unquantified due to the complexity of summer precipitation and diverse influencing factors involved. Nor is there a clear consensus on possible causes of variability in daily extreme precipitation intensities due in part to the restriction of long high-quality daily records with a full spatial coverage over Europe.

Next to the contemporaneous relation between extreme precipitation and atmospheric circulation patterns, the long memory of atmospheric anomalies also creates a delayed influence on extreme precipitation. The links of extreme precipitation with the circulation patterns of the preceding seasons offer considerable benefits for a good seasonal predictability of the relevant hazards for few months ahead. However, there is a relatively low predictability skill for the climate in extra-tropical regions including Europe due to its high variability^16–18^. The skill is worse for inter-annual and decadal variability of precipitation in summer compared to winter^19,20^. The improvement of the state-of-the-art seasonal prediction systems especially for the summer season is a matter of ongoing scientific debate.

To scrutinize the atmospheric processes that transport large amounts of moisture for an extreme precipitation event and to potentially improve seasonal prediction systems considering multiple interacting controls that govern extreme precipitation occurrence, we investigate the concurrent and lagged relationships between extreme precipitation quantiles and atmospheric circulations. We moreover estimate the extent to which decadal variations in extreme precipitation of different seasons can be explained by large-scale atmospheric circulation patterns including NAO, Arctic Oscillation (AO), Southern Oscillation Index (SOI) and Western Mediterranean Oscillation (WeMO) through bivariate and multivariate correlation analyses.

## Results and Discussion

### Decadal variability of extreme precipitation

**Figure 1:**
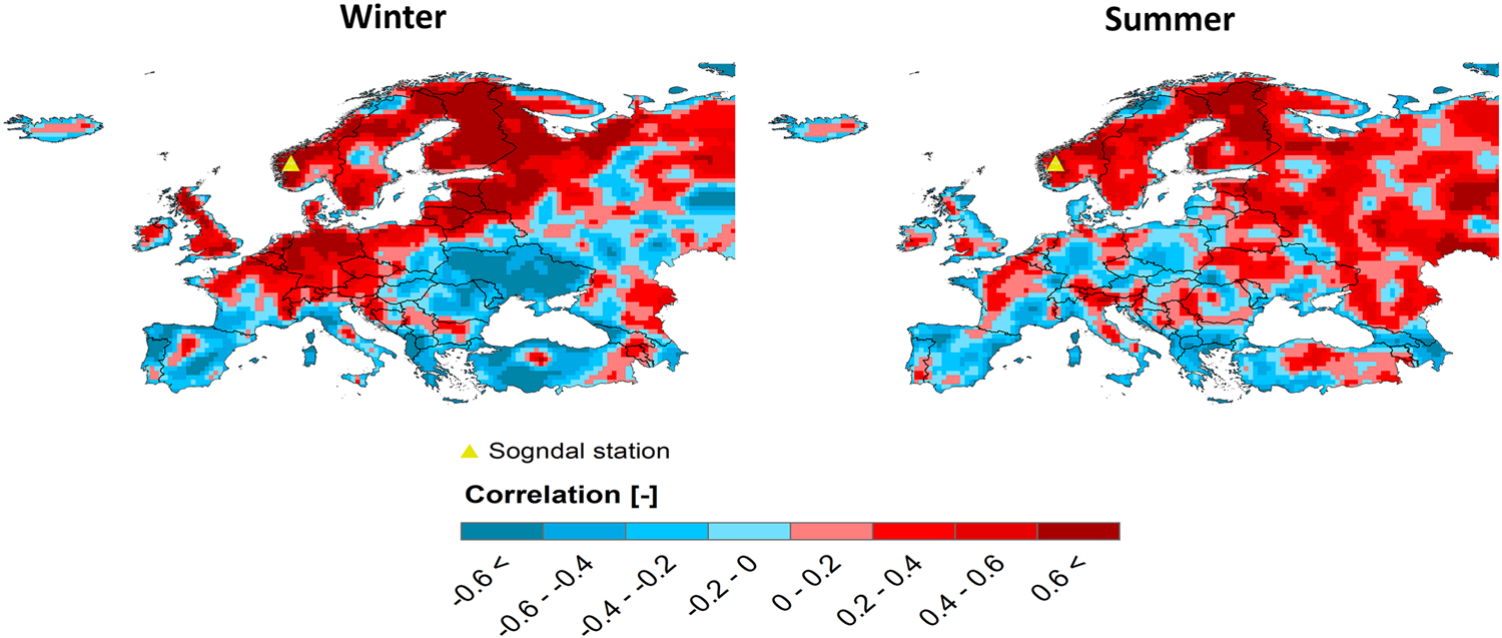
Correlation of daily E-OBS extreme precipitation anomaly between one selected location in North Europe (Sogndal, NO) and the rest of Europe for winter and summer seasons. The maps were generated using the software ArcGIS (version 10) http://www.esri.com/products.

To analyze the decadal variability of European extreme precipitation (defined here as upper 5% of all daily precipitation intensities), next to long raingauge data, we make use of E-OBS dataset with a full and uniform spatial coverage over Europe. However, the gridded data may have limitations for extreme precipitation analysis^21,22^. The comparison between E-OBS and raingauge extreme precipitation anomalies (Figure S2 and Table S2) shows that the E-OBS anomaly generally tracks the decadal variability in the raingauge anomaly (average correlation coefficient of 0.83) with an acceptable bias (≃5% on average). The results reveal that the known underestimation of extreme precipitation by gridded data^3,21^ also leads to an underestimation of the calculated anomaly, but with a smaller magnitude. The bias is, on the whole, larger in summer and autumn than in winter and spring. The bias is moreover smaller for the northern and western Europe compared to the southern and eastern regions, which is attributed to the denser raingauge network in the former regions^23^. After validation of the E-OBS extreme precipitation anomaly, the decadal anomaly was calculated for the entire Europe (Supplementary Movie S1). The significant oscillation high periods have been observed in recent years, associated with several catastrophic floods across Europe^24^ (see Figure S3 for the positive anomalies related to central European floods). Next to the temporal variation of extreme precipitation, there are large spatial differences in the anomalies from north to south and from west to east. This large gradient of extreme precipitation anomaly even within a small region is triggered by the climate effects imposed by the geographical position of ocean and land in the continent and the mountain ranges which act as a low-level air mass barrier^25^. For winter precipitation, which is mainly controlled by large-scale circulations, there are two distinguishable patterns in Europe (Fig. 1). The winter extreme precipitation anomalies in the north and west of Europe closely resemble each other. As opposed to this pattern, there is another pattern for winter extreme anomalies in the south and east of Europe (around the Mediterranean and Black Sea). Figure S4a shows the pattern for raingauges in Norway, Denmark, Netherlands, Belgium and Germany. Compared to winter precipitation, the spatial distribution of summer extreme precipitation is noisier owing to its local and convective nature (Figs 1 and S4b). Nonetheless, there is an anomaly similarity in the Fennoscandia and most of the countries from the former Soviet Union and also in southern Europe. There is a clear anti-correlation between these two groups of anomalies (Figs 1 and S4c).

### Extreme precipitation variability derived by atmospheric circulations

Based on the identified decadal variations of extreme precipitation (>95th percentile), possible causes in terms of atmospheric-oceanic circulations behind the variations are explored for better understanding of the driving forces of weather-related disasters. Unlike most climate variability studies that have examined the relationship between mean precipitation or extreme precipitation frequency and atmospheric circulations using simple correlation methods, the QPM method (see Materials and Methods) was applied here allows in-depth analysis of possible atmospheric drivers of extreme precipitation anomaly. The method acts as a filter to the inter-annual temporal variability, but analyzes the temporal variations of extreme quantiles at (multi-)decadal time scales.

**Figure 2:**
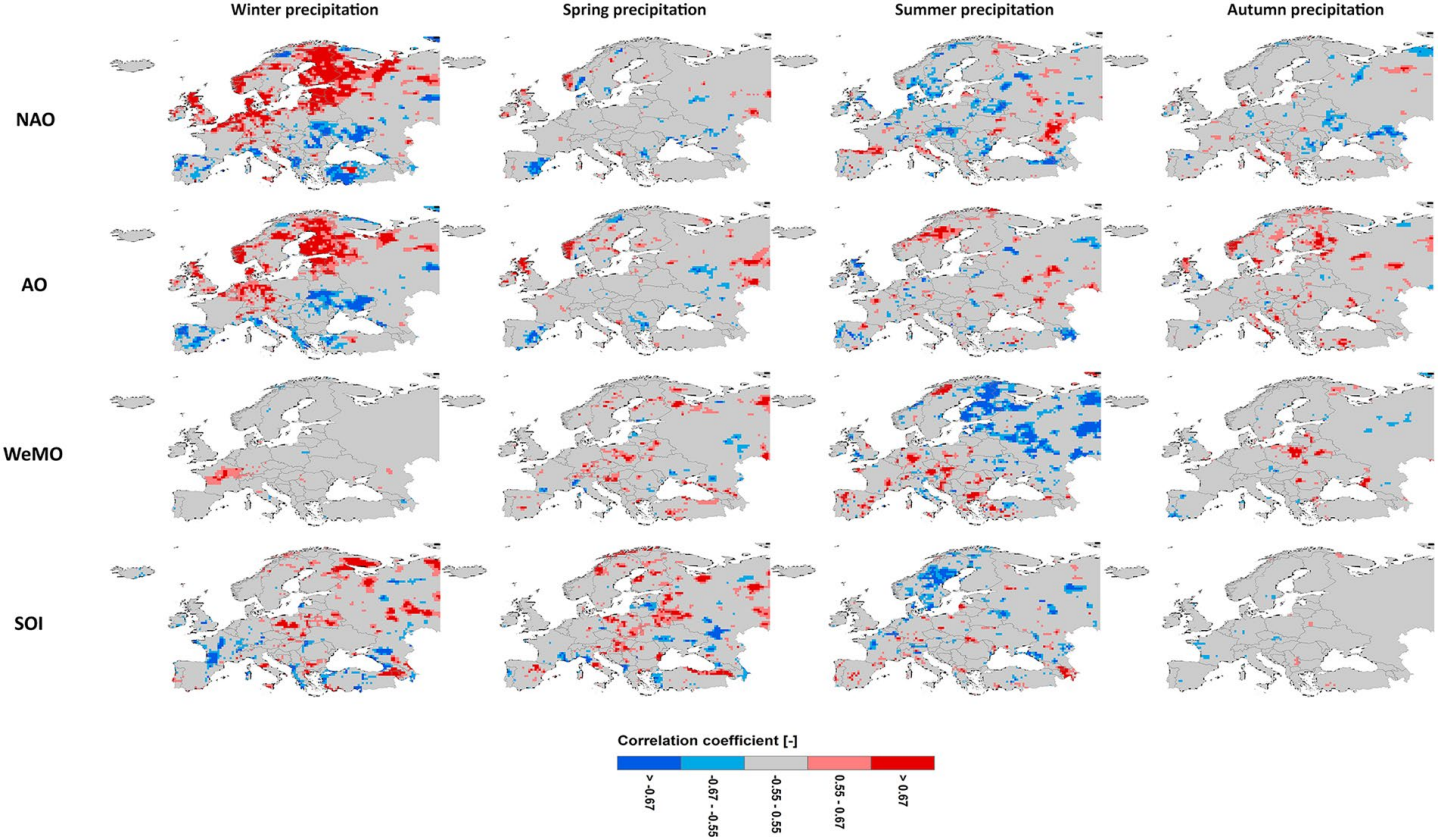
Concurrent correlation between the anomalies of daily E-OBS precipitation extremes and atmospheric circulation patterns for different seasons. Red, pink, gray, light blue and dark blue colors denote significant positive correlations at the 0.05 and 0.10 levels, insignificant correlations, significant negative correlations at the 0.10 and 0.05 levels, respectively. The statistical significance of the correlations is tested according to the t test. The maps were generated using the software ArcGIS (version 10) http://www.esri.com/products.

**Figure 3:**
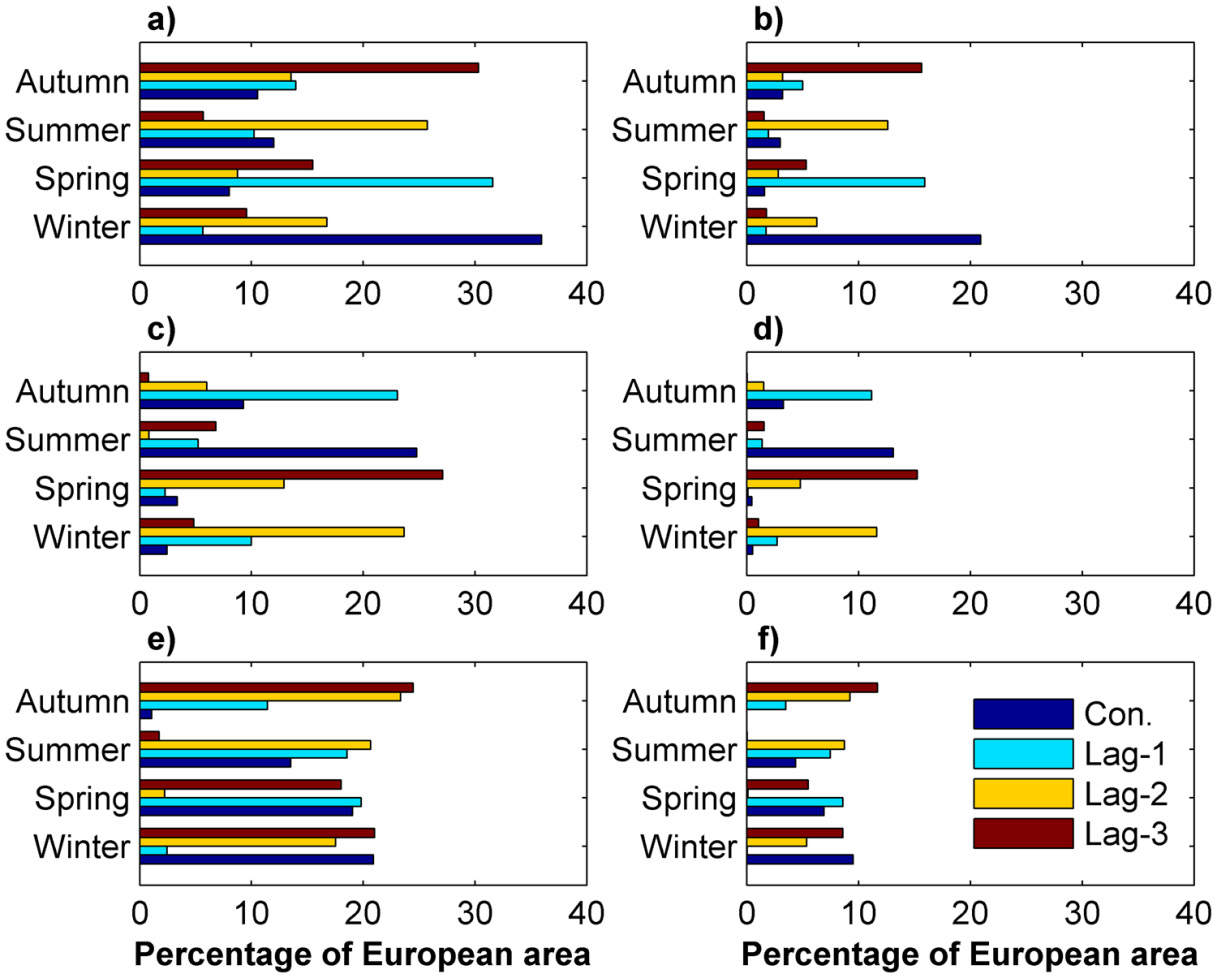
Percentage of European area with a significant bivariate correlation of daily E-OBS precipitation extremes with NAO (**a**,**b**), WeMO (**c**,**d**) and SOI (**e**,**f**) at the 90% (**a**,**c**,**e**) and 95% (**b**,**d**,**f**) confidence levels for the concurrent and lagged relationships. Con.: concurrent correlation; Lag-1: correlation with one season time lag; Lag-2: correlation with two seasons time lag; Lag-3: correlation with three seasons time lag. Autumn, Summer, Spring and Winter denote extreme precipitation anomaly in these seasons.

Figure 2 shows concurrent correlations between the anomalies of daily E-OBS precipitation extremes and atmospheric circulation patterns for different seasons. The North Atlantic Oscillation has a profound influence on winter precipitation extremes in the north and west of Europe, having a significant positive correlation. The sign of correlation changes to negative for southern Europe, creating a dipole-type pattern of winter extreme precipitation anomaly over Europe. The NAO as the major mode of European climate variability varies on the decadal scale due to natural and anthropogenic radiative forcing such as the ones responsible for greenhouse gas increases^26^ as well as internal variability^27^. The European area over which the correlation between the anomalies of extreme precipitation and NAO is significant ranges from 2%(6%) in spring to 21%(36%) in winter for a 0.05 (0.10) significance level (Fig. 3a,b). In order to scrutinize whether the influence of NAO on winter extreme precipitation in Europe in the more distant past was as strong as that in recent years, the relationship between the anomalies of NAO and station winter extremes over a centennial period (116 years) was investigated. The results show a significant positive correlation for many stations in Scandinavia and western Europe (Figure S6). For a shorter 91-year period for which more station data are available, a significant negative correlation is also observed in southern Europe, encompassing Portugal, Spain and southern France (Figure S6). The influence of NAO in the other seasons is much smaller, although there is a significant negative correlation for the summer season in sparse areas over Europe (Fig. 2). Albeit NAO is dynamically the most active during winter, summer NAO can be considered as a weaker counterpart inversely influencing summer extreme precipitation across Europe. An anti-correlation of NAO with summer precipitation over northwest Europe has been reported in the literature^28–30^.

Arctic Oscillation as the hemispheric equivalent of NAO has a very similar (but relatively weaker) influence on winter extreme precipitation in Europe (Fig. 2). The positive periods of AO are associated with more extreme precipitation in the northern half of Europe, while the negative phase brings extreme precipitation to the southern half. The sign of impacts is indeed inverted for the negative phase of AO. A strong AO imprint is not seen in the other seasons, and the regions with a significant correlation are more scattered.

It appears that atmospheric circulations over the Mediterranean Sea (WeMO) have no influence on European extreme precipitation in winter, spring and autumn (Fig. 2). However, there is a dipole-like pattern of the WeMO influence on summer extreme precipitation. With a northeastward shift, the WeMO influence on summer extreme precipitation over Europe is, to some extent, opposite to the influence of NAO and AO on winter extreme precipitation. The linkage of WeMO and the surface climate in the Mediterranean region has previously been postulated^31,32^. The evaporation and atmospheric moisture originated from the Mediterranean Sea have been found to be responsible for summer extreme precipitation and associated flooding over central Europe^33^. The results of this study reveal that this influence has a much larger extent and the atmospheric circulations over the Mediterranean Sea can be considered as the major driver for summer extreme precipitation across the entire Europe.

After analyzing the Atlantic-based climate indices, a question arises as to how much European climate can be influenced by atmospheric circulations over regions that are geographically remote from Europe. To investigate the influence of the Pacific atmospheric circulations on European extreme precipitation variability, the correlations between the anomalies of daily precipitation extremes and the Southern Oscillation Index (SOI) for different seasons are shown in Fig. 2. The influence of SOI on extreme precipitation generally weakens from winter to autumn, showing a seasonally varying teleconnection mechanism between ENSO and European extreme precipitation^34,35^. Winter extreme precipitation anomaly in 21% (10%) of the European area has a significant teleconnection with SOI at the 0.10 (0.05) significance level (Fig. 3e,f). The impact of ENSO on European climate has also been reported mainly for winter and spring in the literature^36–39^.

### Feasibility of developing state-of-the-art seasonal prediction systems

Establishment of relationships between extreme precipitation and atmospheric circulation patterns of preceding seasons may provide information for better understanding of extreme precipitation variability and to skillful seasonal prediction. Such prediction is reflected largely in how much the variance of extreme precipitation can be explained by large-scale circulation patterns of preceding seasons. The connection between the atmosphere and ocean in the oceanic mixed layer provides an opportunity to improve predictions. In fact, due to a strong seasonal cycle of the stability at the base of the oceanic mixed layer, any SLP anomaly can persist below the mixed-layer and influence precipitation in the following seasons^40,41^.

**Figure 4:**
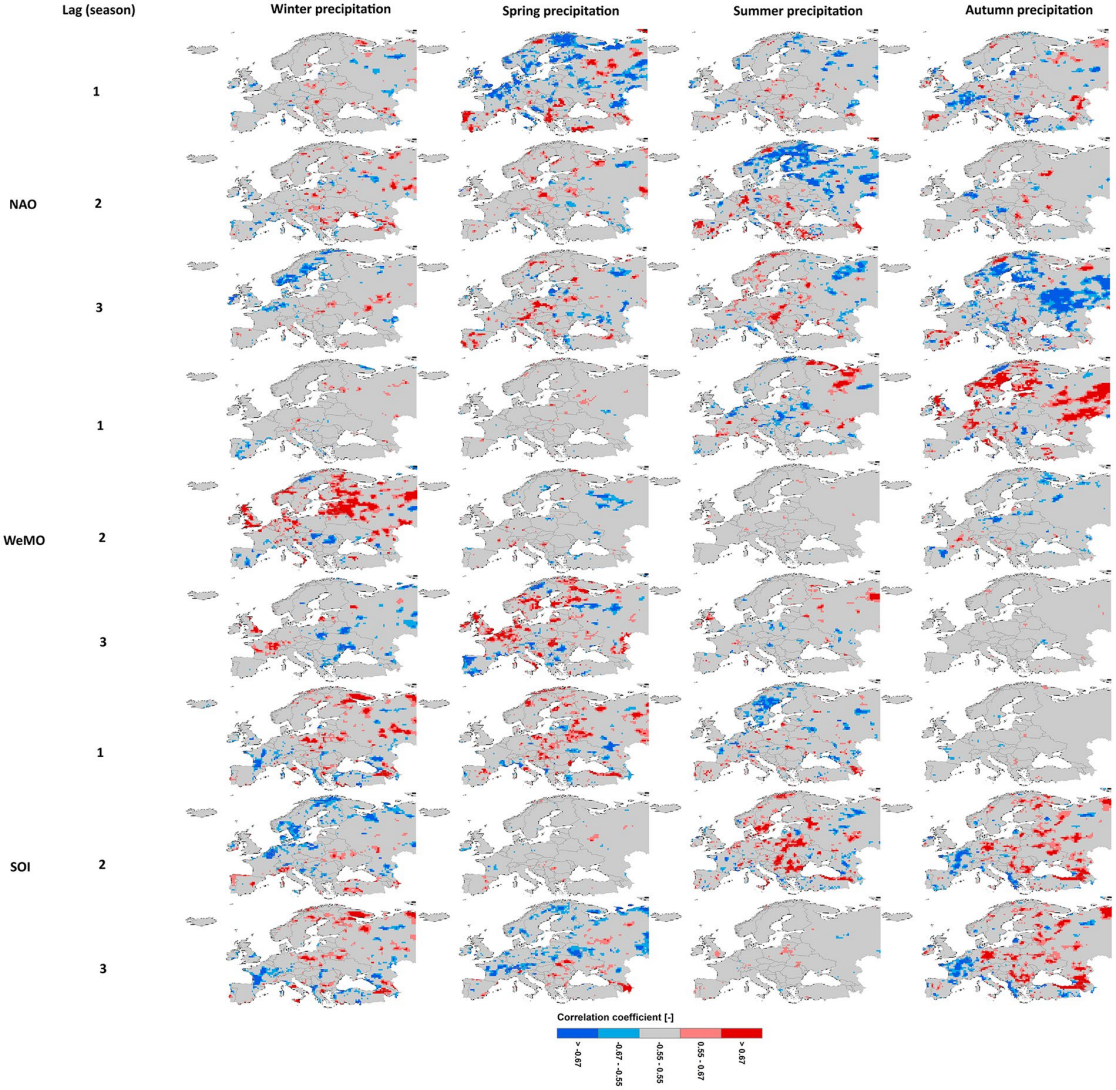
Lagged bivariate correlation of daily E-OBS extreme precipitation anomaly with NAO, WeMO and SOI for different seasons. Red, pink, gray, light blue and dark blue colors denote significant positive correlations at the 0.05 and 0.10 levels, insignificant correlations, significant negative correlations at the 0.10 and 0.05 levels, respectively. The statistical significance of the correlations is tested according to the t test. The maps were generated using the software ArcGIS (version 10) http://www.esri.com/products.

As NAO and SOI have the strongest simultaneous influence on extreme precipitation in winter, and WeMO in summer, the delayed influence of these indices on the extreme precipitation of the coming seasons is investigated (Fig. 4). The results clearly reveal a strong footprint of winter NAO on extreme precipitation of the following seasons. In other words, spring, summer and autumn extreme precipitation is influenced by the preceding wintertime NAO with time lags of one, two and three seasons. The memory provided by the sea-ice, sea surface temperature and snow cover anomalies in the circumpolar regions allows winter NAO signal to influence summer atmospheric circulation in the extratropics^42^. The European area influenced by the preceding NAO reaches its maximum of about 32% (16%) for winter NAO-spring extreme precipitation relationship at the 0.10 (0.05) significance level (Fig. 3a,b). When comparing the maps of simultaneous and lagged correlations, the sign of winter NAO influence in the lagged relationship is somewhat opposite of that in the simultaneous one (Fig. 4). Similar to the NAO influence pattern, summertime WeMO exerts a significant control on extreme precipitation of the following seasons (Fig. 4). The impact pattern of summer WeMO on simultaneous extreme precipitation is, to some extent, reversed for the lagged relationship. The influenced area by summer WeMO increases with increasing time lag: 23% (11%), 25% (13%) and 27% (15%) for time lags of one, two and three seasons for the 0.10 (0.05) significance level (Fig. 3c,d). Similarly, winter SOI has a controlling influence not only on winter precipitation extremes, but on the extremes in the following seasons (Fig. 4). A recent study also reported the winter ENSO forcing of spring European climate^43^. Our findings corroborate those previous results, but also fortify it for the other seasons. Our results indeed show the strongest linkage of European extreme precipitation for different seasons to the preceding wintertime SOI. The influenced areas by wintertime SOI range from 20% (10%) for spring precipitation to 25% (12%) for autumn precipitation for the 0.10 (0.05) significance level (Fig. 3e,f). These results offer potential predictability of European extreme precipitation on a seasonal scale and in general mark a significant step forward, given the large climate variability and a limited amplitude of the atmospheric response over the Atlantic-European sector^44,45^. Although investigation of the underlying physical mechanisms responsible for such a long memory of atmospheric anomalies is beyond the scope of this study, it is anticipated that the following two consecutive processes are in play: the persistence of the atmospheric winter ENSO signal in the stratosphere (after upward propagation of Rossby waves from the troposphere to the stratosphere) considering its slow evolution, and the atmosphere–ocean mixed layer interaction in the North Atlantic^43,46^. In fact, the footprint of the wintertime ENSO signal is retained in the ocean for a long time and then transferred back into the atmosphere in the following seasons through the sea–air interaction^43,47,48^. In summary, the stratosphere appears to act as a bridge to link the tropical Pacific Ocean variability to the North Atlantic. Seasonal prediction systems on the European climate may benefit from this linkage^49^.

**Figure 5:**
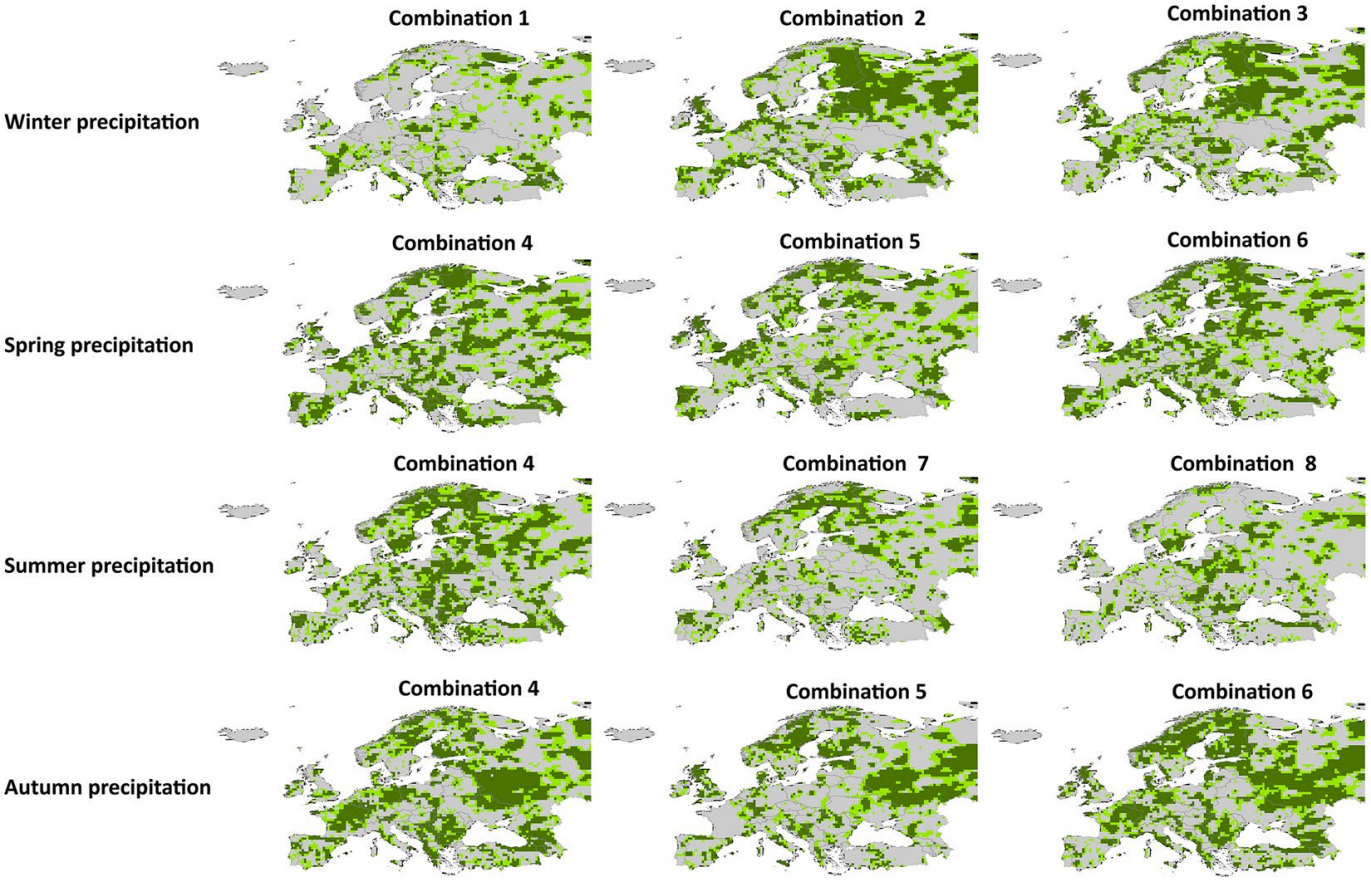
Multiple correlation coefficients between the anomalies of daily E-OBS precipitation extremes and climate indices for different seasons. Significantly influenced European areas are shown in light and dark green colors for the 0.10 and 0.05 levels, respectively. See Table S3 for the climate indices used for each combination. The maps were generated using the software ArcGIS (version 10) http://www.esri.com/products.

When examining the field significance of the bivariate correlations, there is no field significance in the bivariate correlation maps at the continental scale (Figure S7), although the influence of the climate indices is noticeable in some region (at the regional scale). To combine the partial influence of each of the climate indices on European extreme precipitation, a multivariate linear regression approach was employed. Based on the results of bivariate correlation, three combinations of the most effective indices were correlated with extreme precipitation in each season (Table S3). The larger the influenced European area and the higher the correlation coefficients, the greater the explained variance in extreme precipitation. Combination 2 with summer NAO and summer WeMO as inputs presents the best ability to explain the variance of winter extreme precipitation, while Combination 1 merging summer NAO and spring SOI effects is by far the worst combination for the winter season (Fig. 5). This plainly shows a higher forecasting skill for winter extremes for the combinations including summer WeMO. Using Combination 2, the influenced European area goes up to 33% for a significance level of 0.05 (Figure S8). For summer extreme precipitation, Combination 4 with inputs of winter NAO and winter SOI clearly shows a better performance compared to Combinations 7 and 8, significantly (at the 0.05 level) affecting about 32% of the European area. This implies the higher skill of the summer extreme precipitation for the combinations excluding the decaying signal of autumn WeMO. Combinations 4 and 6 perform the best for the spring and autumn extreme precipitation respectively, whereas Combination 5 with winter NAO and summer WeMO appears to be the worst combination for both seasons. The influenced European area at the 0.05 significance level for Combination 4 of spring precipitation and Combination 6 of autumn precipitation are 35% and 40%, respectively. Generally, the spatial pattern of the influence for different combinations is different, depending on the atmospheric indices involved in the combinations. The influenced area for the winter and summer seasons is smaller than that for the transitional seasons (spring and autumn). Nonetheless, all multiple correlation maps have field significance (not shown), suggesting that extreme precipitation being modulated by a combination of large-scale atmospheric circulations. In fact, the effect of each atmospheric circulation pattern is small to be confidently distinguished from noise, but it is large and distinguishable when combined.

The multivariate linear relationships are evaluated using station precipitation of a longer centenary records. The results (see Figure S9 for the results for some selected stations) show high predictive skill of the combinations over different hindcast periods and confirm the combined effects of the selected circulation patterns on extreme precipitation anomaly over Europe. The results also indicate that the combined influence of atmospheric circulation patterns is not always additive and they could also counteract each other for some regions and time of year. Next to the obtained influence of atmospheric-oceanic circulations leading to moisture transport from the oceans to the European land, regionally recycled moisture (i.e., recycling of evaporation from land surfaces rather than from over the oceans) contributes a substantial component of precipitation for Europe particularly in summer^50,51^. This is more the case for eastern Europe where about 40–60% of precipitation comes from moisture recycling^50,52^.

### Final Remarks

Our analysis provided an evidence for concurrent and delayed influences of the atmospheric circulations over the North Atlantic and Pacific oceans and the Mediterranean Sea on European extreme precipitation variability. The results are especially important for summer extremes for which the driving forces have been less quantified. Specifically, summer extreme precipitation is influenced by concurrent summer WeMO forcing and the preceding winter NAO and SOI forcing. In fact, a combination of the partial influence of large-scale atmospheric circulations modulates extreme precipitation variability over Europe. The enhanced understanding of the multiple interacting atmospheric processes controlling the occurrence of extreme precipitation and flooding obtained here would be immensely beneficial for improving the state-of-the-art seasonal prediction systems and for compensating the inaccuracy of numerical weather forecast models for heavy precipitation events^53,54^. In turn, such improved predictability skill could be potentially useful to develop seasonal risk outlooks for mitigation policy definition, adaptation planning, disaster preparedness and budgetary planning to reduce the risk of fund depletion due to unexpected payouts for flood disasters.

## Materials and Methods

### Data

Daily precipitation data from the gridded E-OBS dataset (version 13.1) for the period 1950–2015 are used (Table S1). The E-OBS dataset was originally developed as part of the ENSEMBLES (http://ensembles-eu.metoffice.com/) and EURO4M (http://www.euro4m.eu/) projects and later on it has been regularly updated by including more stations under the UERRA project (http://www.uerra.eu/). This gridded dataset was created from measurements of more than 10,000 stations using kriging interpolation method^55^. We also make use of long precipitation time series from 263 (187) European stations for the period 1925–2015 (1900–2015).

To explain the anomalous behavior of extreme precipitation over Europe, it is connected to several climate indices (atmospheric circulation patterns). As some of climate indices are not available at the daily time scale, we developed them based on the SLP difference between two specific locations of each index (e.g., Tahiti and Darwin for SOI) using SLP data from 20th Century Reanalysis V2c^56^ for different seasons. The seasons are defined as follows: December, January and February as winter, March, April and May as spring, June, July and August as summer and September, October, and November as autumn. The climate indices used are:

- North Atlantic Oscillation (NAO): This index is defined as the normalized SLP difference between the Azores high pressure and the Icelandic low pressure. Mean daily data of the NAO index for the period 1950–2015 were obtained from the NOAA Climate Prediction Center. For the older time periods, daily NAO index series back to 1850 developed by Cropper *et al*.^57^ are used.
- Arctic Oscillation (AO): This index is constructed by projecting the daily 1000 mb height anomalies poleward of 20°N onto the leading the empirical orthogonal function (EOF) of the monthly mean 1000 mb height during 1979–2000 base period. Daily mean data of the AO index for the period 1950–2015 were obtained from the NOAA Climate Prediction Center.
- Southern Oscillation Index (SOI): We developed daily SOI data using reanalysis SLP data as the SLP difference between Tahiti and Darwin normalized based on the period 1981–2010.
- Western Mediterranean Oscillation (WeMO): We developed daily WeMO data using reanalysis SLP data as the SLP difference between Cádiz-San Fernando (Spain) and Padua (Italy) normalized based on the period 1981–2010.

More information about these teleconnection patterns can be found in the literature^58–60^.

### Quantile perturbation method (QPM)

The QPM^61,62^ is used to analyze decadal anomaly in extreme precipitation for different seasons. The QPM takes into account both the frequency of occurrence and intensity of extreme precipitation by comparing extremes from the full time series with the ones from a time slice (a 10-year period in our case) of the full time series (sub-series) with the same exceedance probability. To calculate an overall anomalies for each sub-series, the anomaly above the selected threshold for extremes, by assuming fairly constant anomaly of higher quantiles, are averaged:

1 $$PF=\frac{\sum _{k=1}^{n}\frac{{X}_{{S}_{k}}[p]}{{X}_{{F}_{k}}[p]}}{n}$$

where PF is the perturbation factor or anomaly (unitless), X_S_ and X_F_ are the extreme precipitation with the exceedance probability p based on an empirical distribution for the sub-series and the full time series respectively, and n is the number of extreme precipitation events between a certain threshold and the maximum value. The anomaly values greater (less) than one indicate positive (negative) anomaly, pointing to higher (lower) quantile values in the sub-series (10-year block) than in the full time series. The 95th percentile of all precipitation data of the whole available period for each station/grid point separately is selected as the threshold for extremes in this study. Figure S1 shows the spatial distribution of European precipitation for the selected threshold. The sensitivity analysis of the results to changing the exceedance probability for defining extremes from the 95th percentile to the 99th and 90th percentiles shows that the results are not sensitive to the selected threshold, approving the assumption for fairly constant anomaly of higher quantiles.

In the case of mismatch between the exceedance probability of values in the sub-series and the full time series, the values for the full time series are obtained using an interpolation from the two adjacent values with the closest exceedance probability. To compare the extremes between any possible sub-series and the full time series, the time slice in the sub-series is moving with a sliding window of one year from the beginning towards the end of the full series. Finally, the significance of the anomalies is tested using a nonparametric bootstrapping method.

### Correlation analysis

#### Significance testing

The correlation between the anomaly of extreme precipitation and climate indices is investigated using the correlation method. The anomaly series of extreme precipitation and climate indices are linearly detrended before the correlation analysis to ensure that the correlation only reflects shared time series variation at the (multi-)decadal scale independent of any underlying trend. The existence of autocorrelation in the anomaly series calculated by the QPM will increase the probability of rejecting the null hypothesis of no significant correlation between the anomalies of extreme precipitation and climate indices, while the null hypothesis is true. In order to take autocorrelation into account, either the autocorrelation structure of the time series is filtered out or statistical method is modified. In this study, the effective sample size (equal to the number of block periods) is used to correct the number of degrees of freedom for the significance testing of the correlation coefficient using t test:

2 $$t=\frac{r\sqrt{(\frac{n}{l})-2}}{\sqrt{1-{r}^{2}}}$$

where r is the correlation coefficient, n is the actual sample size of the data and l is the length of the sub-series for the QPM analyses which is equal to 10 in this study.

#### Multiple correlation

Atmospheric circulation patterns, in reality, do not affect extreme precipitation individually, but they may do jointly. To investigate the joint influence of atmospheric circulation patterns on extreme precipitation anomaly, multiple correlation coefficient (R) is used. For small sample sizes, R may be a biased estimate of the population multiple correlation coefficient. The main bias of R is that it will increase by adding more predictors to the model no matter if the predictor is really effective or not, leading to high R values for models with more predictors. In fact, high R values can be obtained due to including random noise to the data and the model’s overfitting. The R statistic is modified to take into account the number of predictors in the model and represent a fairly unbiased correlation coefficient by

3 $${R}_{adj}=\sqrt{1-\frac{(1-{R}^{2})-(n-1)}{n-k-1}}$$

where k is the number of predictors and n the number of data. ${R}_{adj}$ is between 0 and 1 and always less than or equal to R^63,64^.

The existence of collinearity inflates the variance of the regression coefficients in multiple correlation method and leads to a biased result. Collinearity exists when pairs of predictors are highly correlated. To avoid collinearity, independent climate indices as predictors need to be included in the multiple regression model. The variance inflation factor (VIF) is used as an indicator of collinearity^65,66^.

4 $$VIF=\frac{1}{1-{R}_{adj}^{2}}$$

VIF > 10 is an indication of the presence of collinearity. The lower values of 5 or 4 have also been recommended as the maximum VIF value in the literature^67,68^.

#### Field significance

For the correlation analysis between the anomaly of extreme precipitation over grid points and climate indices, it is assumed that the variability of extreme precipitation at different locations is independent. This assumption may not be valid as variability in a geographic region tends to be correlated with variability in an adjacent region. To test this assumption, several methods have been proposed, each with its pros and cons^69–71^. In this study, the field significance of the correlations is determined in a Monte Carlo framework as in Livezey and Chen^69^. The time sequence of climate index anomaly is randomly reordered. The correlation coefficient between extreme precipitation anomaly and the randomly reordered climate index anomaly is computed and then the European area over which the correlation is significant at the 5% level is obtained. This is based on the fact that the random resampling of the climate index anomaly breaks any possible connection between the climate index and the extreme precipitation. This procedure is repeated a large number of times (1000 times in our case), each time by using a new random resampling. The result is a sample of 1000 percentage values of European area with significant correlations. There is a field significance in the correlation maps if the percent of European area with significant correlations at the 5% level between the anomalies of extreme precipitation and the real climate indices lies in the upper 5% tail of the area obtained by using random reordered time series. The method is modified for the multiple correlation, each time by randomly resampling of the time series of the two inputs for each combination.

### Data availability

The E-OBS and raingauge data are freely available at the website of the European Climate Assessment and Data (http://www.ecad.eu/download/ensembles/download.php). The 20th Century Reanalysis data provided by the NOAA/OAR/ESRL PSD, Boulder, Colorado, USA, are publicly available from their Web site at http://www.esrl.noaa.gov/psd/.

## Electronic supplementary material

Movie S1

Supplementary Information
