# Supplementary material for: Lagged influence of Atlantic and Pacific climate patterns on European extreme precipitation
Source: Sci Rep. 2018 Apr 10;8:5748. doi: 10.1038/s41598-018-24069-9 (PMC5893627; doi:10.1038/s41598-018-24069-9)
Supplement: Supplementary file 2 — Supplementary Information [file 41598_2018_24069_MOESM2_ESM.docx]

Supplementary Materials

**Lagged influence of Atlantic and Pacific climate patterns on European extreme precipitation**

**Hossein Tabari, Patrick Willems**

Correspondence to: hossein.tabari@kuleuven.be

**This PDF file includes:**

Supplementary Method

Supplementary Text

Figures S1 to S9

Tables S1 to S3

Caption for Movie S1

Supplementary References

**Other Supplementary Materials for this manuscript includes the following:**

Movie S1

Supplementary Method

**Wavelet coherence**. In addition to general correlation between two time series, it is useful to investigate the co-variation of the periodicities of two time series using the wavelet coherence method^1,2^. The coherence coefficient can be considered as a localized correlation coefficient in time-frequency space^3^ and is given by

$R^{2}(s,t)=\frac{\left|S\left(s^{-1}W_{XY}(s,t)\right)\right|^{2}}{S(s^{-1}\left|W_{X}(s,t)\right|^{2}).S(s^{-1}\left|W_{Y}(s,t)\right|^{2})}$ (1)

where $W_{X}\left( s,t \right)$ and $W_{Y}\left( s,t \right)$ are the wavelet transforms of the two time series at the scale *s* and the time *t*,$W_{XY}\left( s, t \right)$ is the cross-wavelet power and *S* is a smoothing operator. The cross-wavelet power spectrum is defined as

$W_{XY}(s,t)=W_{X}(s,t)W_{Y^{*}}(s,t)$ (2)

where $W_{Y^{*}}\left( s, t \right)$ is the complex conjugate of $W_{X}\left( s, t \right)$. The operator *S* contains the smoothing along the wavelet scale $(S_{scale})$ and time $(S_{time})$:

$S(W)=S_{scale}(S_{time}(W(s,t)))$ (3)

$S_{scale}$ and $S_{time}$ are calculated using different relationships for different wavelet functions. For the Morlet wavelet which is used in this study, they are defined as

$S_{time}(W)\left|_{s}\right.=(W(t,s).c_{1}^{\frac{-t^{2}}{2s^{2}}})\left|_{s}\right.$ (4)

$S_{scale}(W)\left|_{t}\right.=(W(t,s).c_{2}\prod(0.6s))\left|_{t}\right.$ (5)

where c_1_ and c_2_ are normalization constants and is the rectangle function^4,5^.

Supplementary Text

**Pros and cons of using gridded precipitation data.** This study benefits from the E-OBS gridded data with a full coverage over Europe and acceptable spatial resolution. This is important to find regional patterns of coherent extreme precipitation variability. The E-OBS data also provide precipitation estimates in data-sparse regions which are away from raingauges. Yet, the data represent an areal average over a grid box rather than point measurements like raingauges which may pose a bias to decadal anomaly of extreme precipitation. A research question here is how much the smoothing of precipitation extremes in the interpolated E-OBS dataset affects the pattern and magnitude of decadal anomaly. Figure S2 shows a comparison between the anomaly of raingauge and E-OBS extreme precipitation for some selected locations in Europe. As can be seen, the anomaly pattern from the E-OBS dataset generally follows the one from raingauge data, although an underestimation by the E-OBS data is observed particularly for the southern and eastern regions with sparser raingauge network.

**Extreme precipitation anomaly leading to floods: central Europe floods.** Central Europe has been hit by several devastating floods in recent summers, of which the most destructive ones were those in summer 2002 and 2013. These two events affected the Czech Republic, Austria, Germany, Slovakia, Poland, Hungary, Romania, Croatia and Russia, causing tens of fatalities and damage of billions of euros. During these events, some places received a huge amount of precipitation equal to their normal monthly precipitation in only one or two days. Figure S3 shows extreme precipitation anomaly for some selected stations in central Europe. The statistical significance of the anomalies is tested by the nonparametric bootstrapping method at the 95% confidence level. The anomalies between the upper and lower limits of the confidence interval (the region of acceptance of the null hypothesis) are considered insignificant, while those outside the region of acceptance of the null hypothesis are defined as statistical significant. There is a clear indication of clustering of precipitation extremes during recent summers in central Europe, with a significant positive anomaly after the 2000s.

**Relationship analysis to circulation patterns as a function of time and frequency.** In addition to correlating the anomalies of extreme precipitation and large-scale patterns, their 99th percentiles were also correlated as a function of frequency and time using the Wavelet coherence method. Figure S5 shows the relationship between the 99th percentiles of winter precipitation and NAO at Uccle and Kiev stations using the Wavelet coherence approach. In these plots, blue-to-yellow color code is associated with the coherence power between the two series, with yellow color showing the cone of influence (COI) or the highest coherence power. The thick black contours represent the 5% significance level. Arrows identify the phase relation between two series, with a right-pointing arrow indicating an in-phase relation (or correlation) and a left-pointing arrow indicating an anti-phase relation (or anti-correlation), and arrows pointing up or down mean a lag between the two series. The results show a continuous influence of NAO on winter extreme precipitation at Uccle station (Belgium) at a 35-year period which is statistically significant around 1960. There is a similar cyclic influence of NAO on winter extremes in Kiev (Ukraine), but in opposite sign and stronger.

**Reanalysis of the linkage to circulation patterns using long station records.** To test the robustness of our conclusion for the relationship between E-OBS extreme precipitation and atmospheric circulations, the correlation between the anomalies of precipitation extremes and the climate indices was reanalyzed using long station records to see whether accidental correlations in shorter gridded data degenerate and to check whether the possible bias in the gridded E-OBS extreme precipitation anomaly influences the relationships. Because stations with long precipitation data are mostly concentrated over western and northern Europe, the relationship can be examined only for the circulation patterns influencing these regions. The North Atlantic Oscillation is one of those patterns whose relationship with station precipitation extremes during boreal winter is shown in Figure S6. The correlation analysis was performed for two long periods: a 91-year period with 263 raingauges and a 116-year period with 187 raingauges. The results for the 20th century raingauge precipitation data confirm the linkage obtained using shorter E-OBS dataset. For the 116-year period (1900-2015), a significant positive correlation is mostly observed for the stations located in Belgium, the Netherlands, Germany, Denmark and Norway. The shorter period of 91 years (1925-2015) enables us to have a larger number of raingauges especially in southern Europe. The results for the shorter period reveal a significant negative correlation mainly for the stations located in Spain, Portugal and southern France.

**Combined influence of climate indices.** In order to check the feasibility of developing models for the state-of-the-art seasonal forecasting systems, the most effective atmospheric indices for each season were selected. Based on the results in Figure 3, apart from the concurrent influence, European winter extreme precipitation is mainly controlled by three indices of summer NAO, spring SOI and summer WeMO. Hence, three combinations of these indices were used for winter extreme precipitation: Combination 1 with inputs of summer NAO and spring SOI; Combination 2 with inputs of summer NAO and summer WeMO; Combination 3 with inputs of spring SOI and summer WeMO (Table S2). Next to considering the most effective predictors, their independency is another prerequisite for multivariate analysis. The statistical analysis shows that the climate indices of the winter combinations are statistically independent (no collinearity) with variance inflation factor (VIF) equal to 1.14, 1.34 and 1.00 for Combinations 1, 2 and 3, respectively. A similar procedure is used for combining the influence of climate indices for the other seasons.


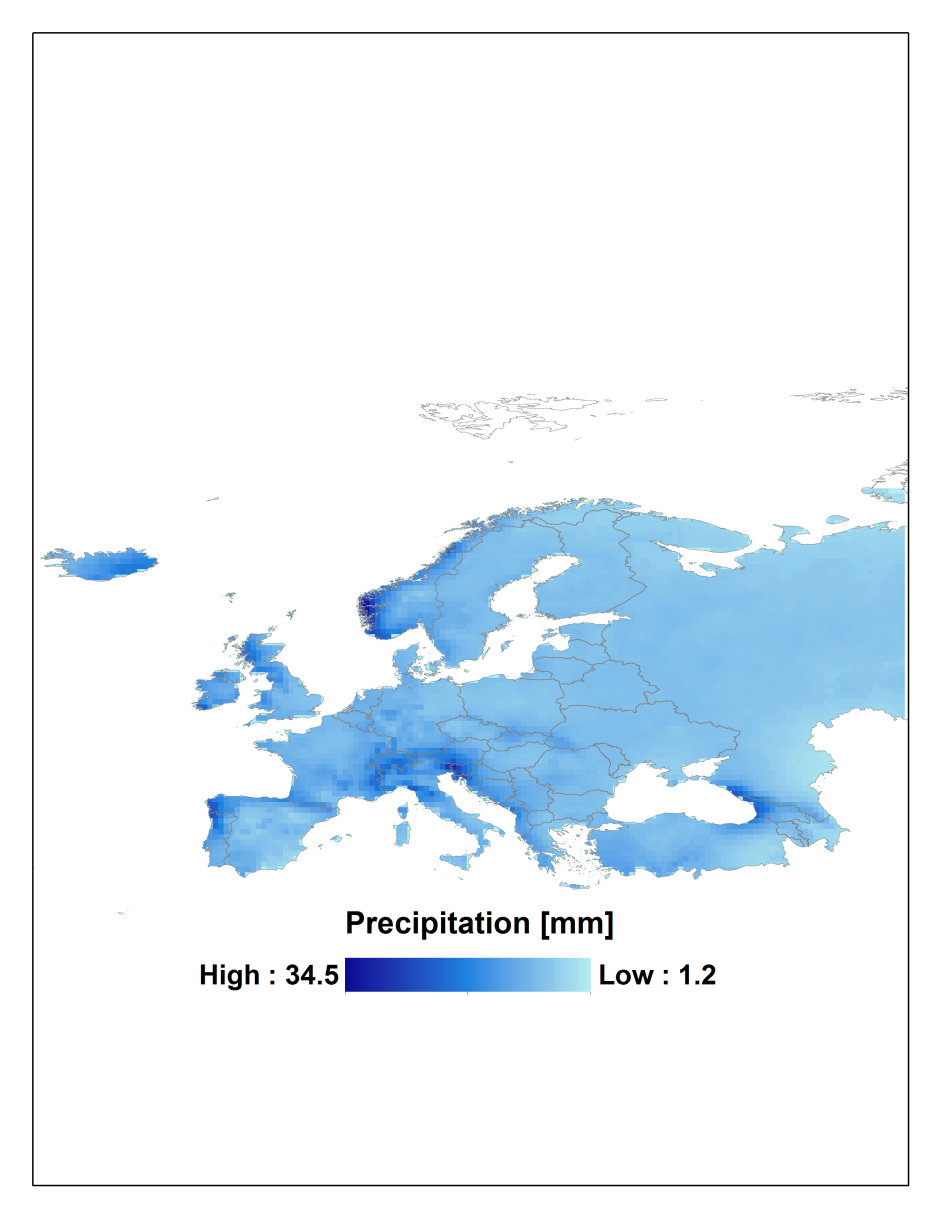


**Figure S1.** Spatial distribution of the 95th percentile of European precipitation for the period 1950-2015. The map was generated using the software ArcGIS (version 10) <http://www.esri.com/products>.


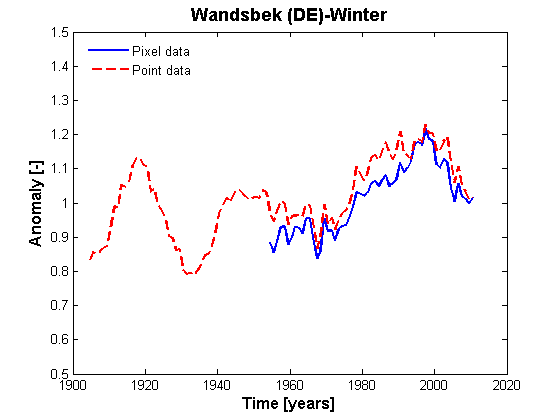

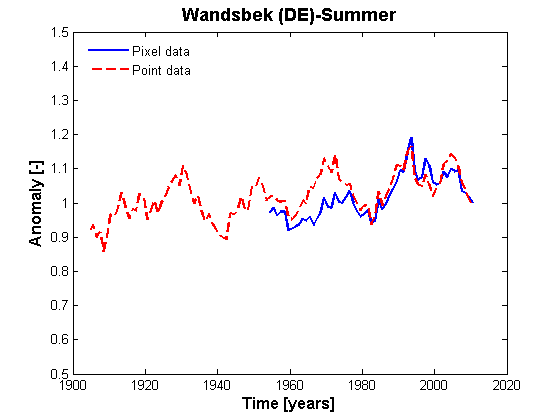

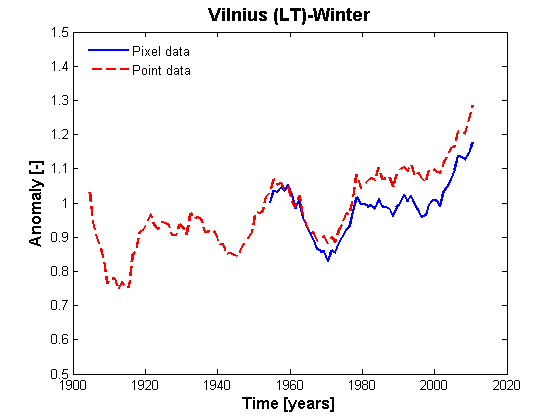

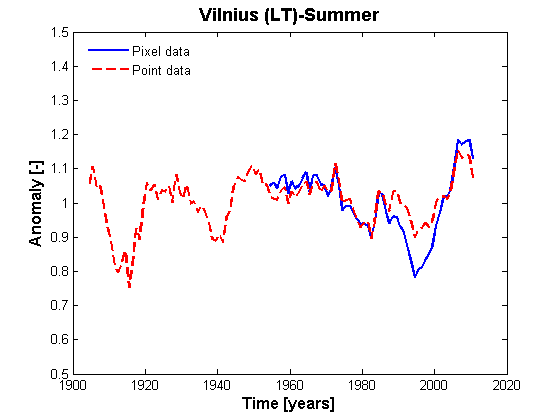

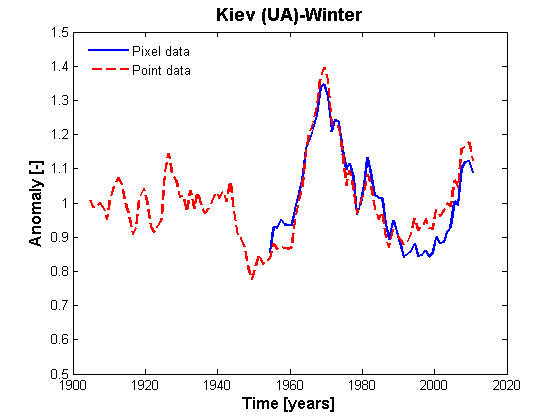

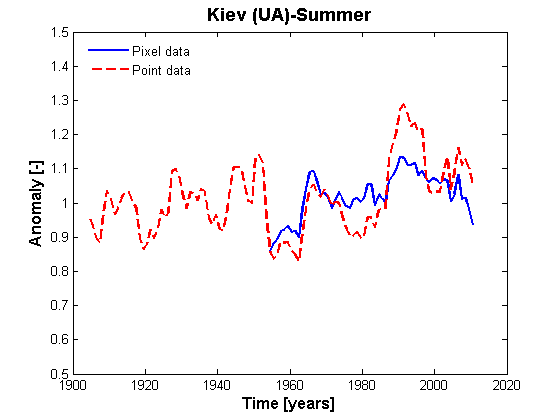


**Figure S2.** Comparison of decadal extreme precipitation anomaly using point (raingauge) and interpolated pixel (E-OBS) data for some selected locations, for the winter (left column) and summer (right column) seasons.


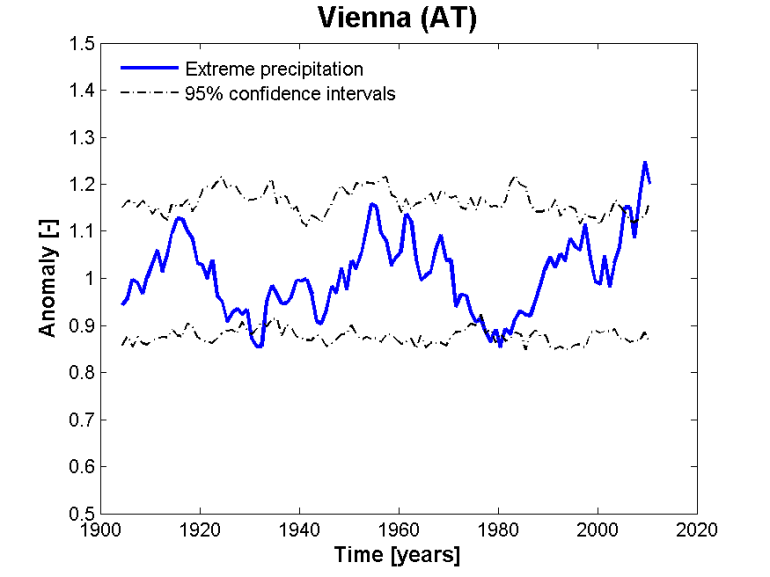

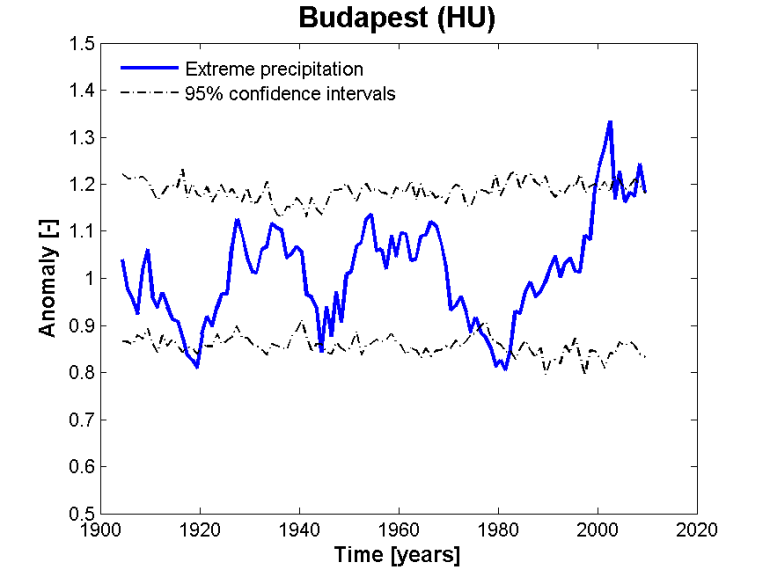


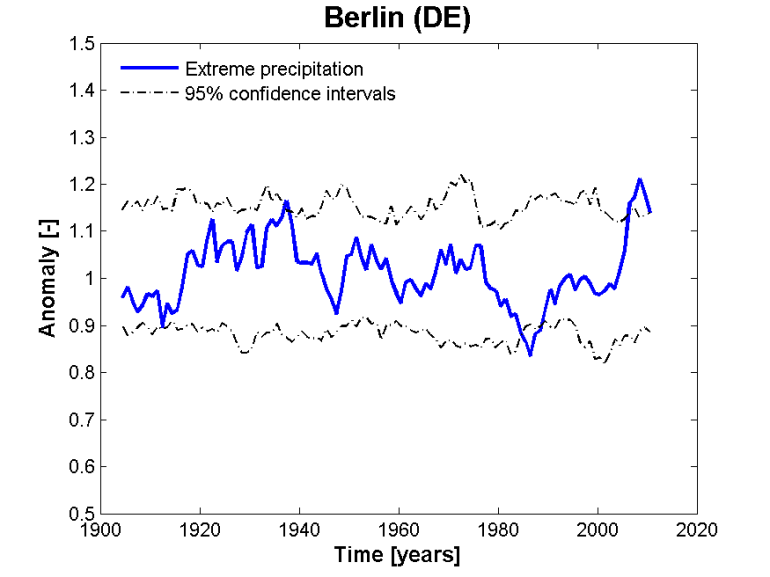

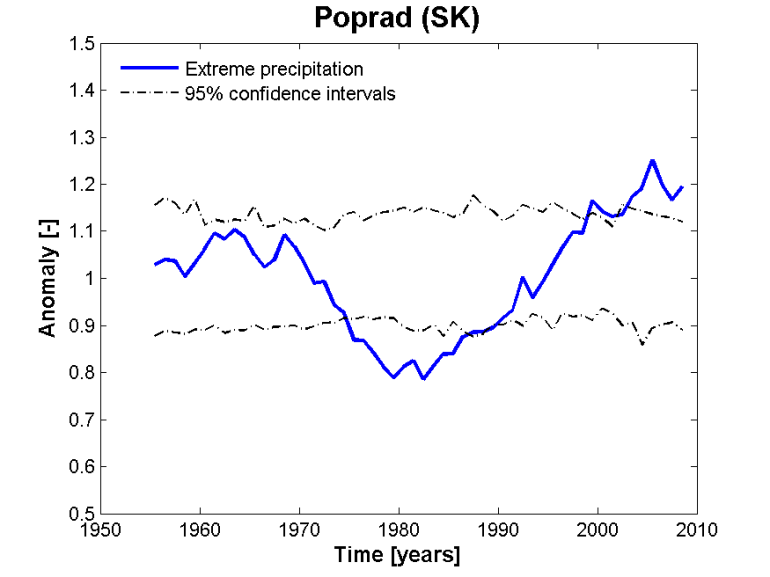


**Figure S3.** Extreme precipitation anomaly along with 95% confidence intervals for some selected stations affected by recent devastating floods in central Europe. Significant positive anomalies at the 95% confidence level are highlighted by dashed red circle.

| **a)** | **b)** |
| --- | --- |
| 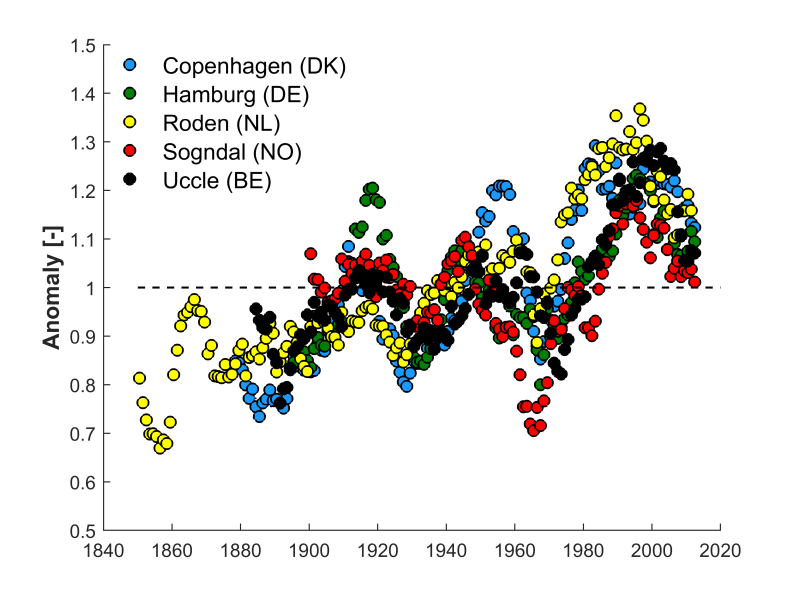 | 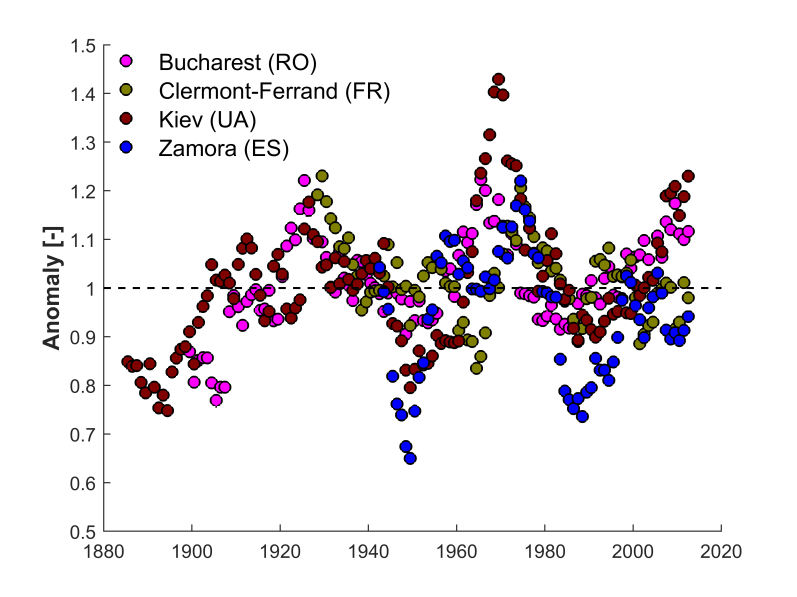 |
| **c)** | |
| 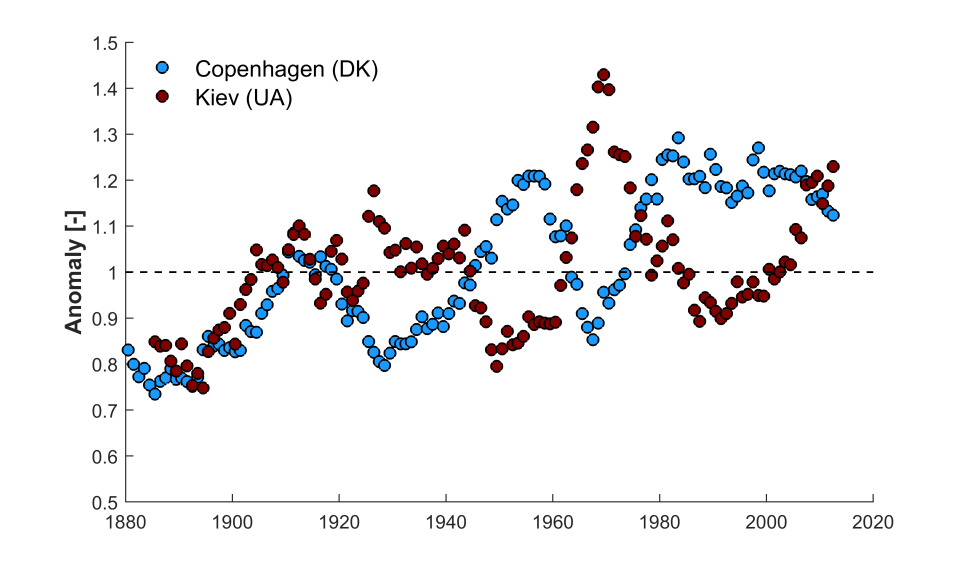 | |

**Figure S4.** European extreme precipitation anomaly for the winter season: a) Anomaly for some selected raingauges in North and West Europe (Region 1: R1); b) Anomaly for some selected raingauges in South and East Europe (Region 2: R2); c) Anomaly comparison between R1 and R2.

**
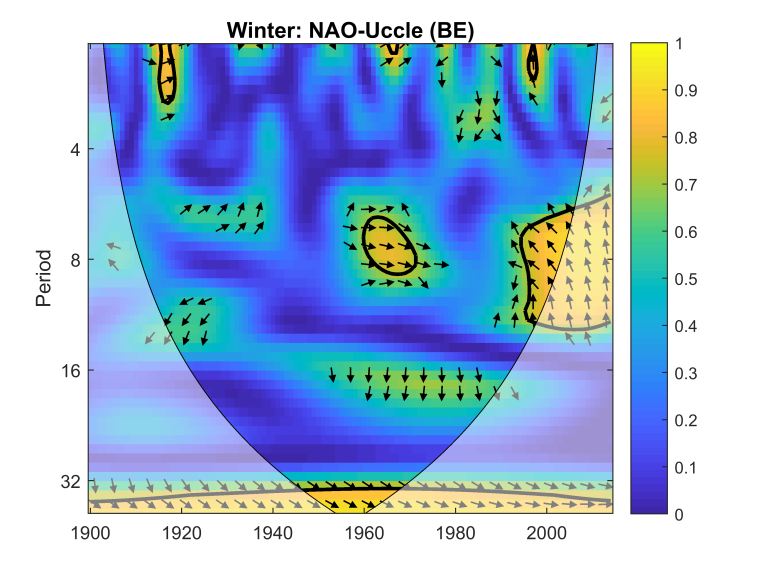

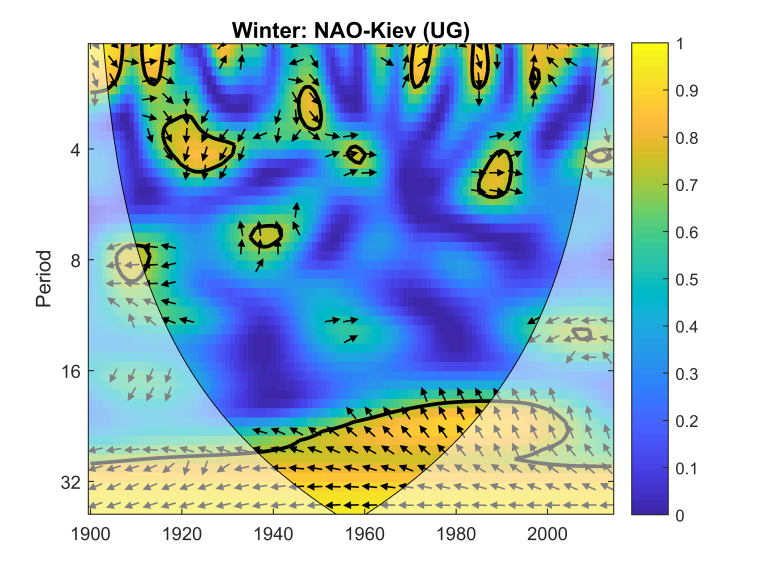
**

**Figure S5.** Wavelet coherence between the 99th percentiles of precipitation and NAO at Uccle (Belgium; left plot) and Kiev (Ukraine; right plot) stations.


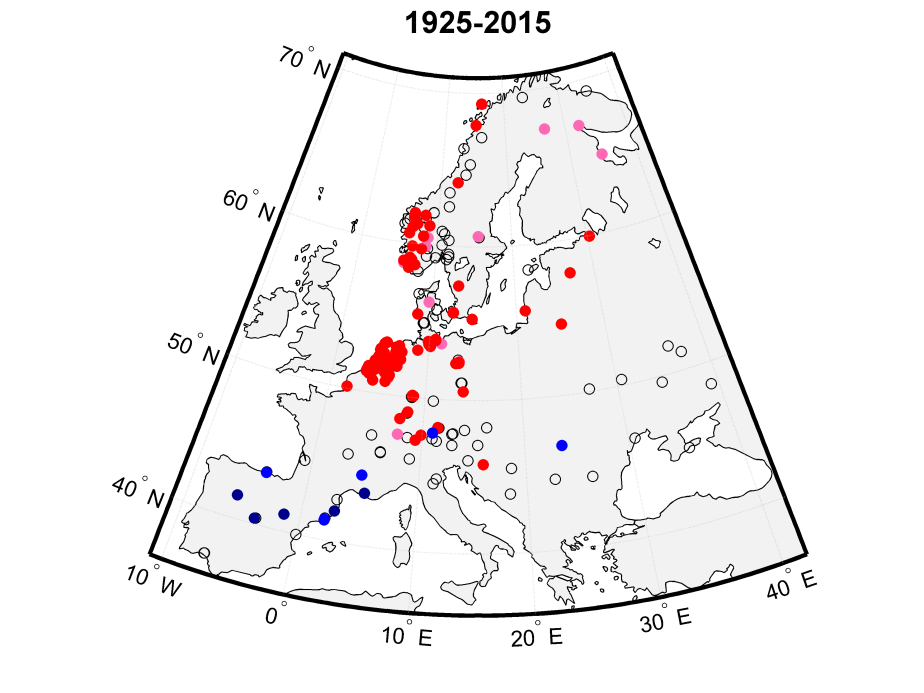

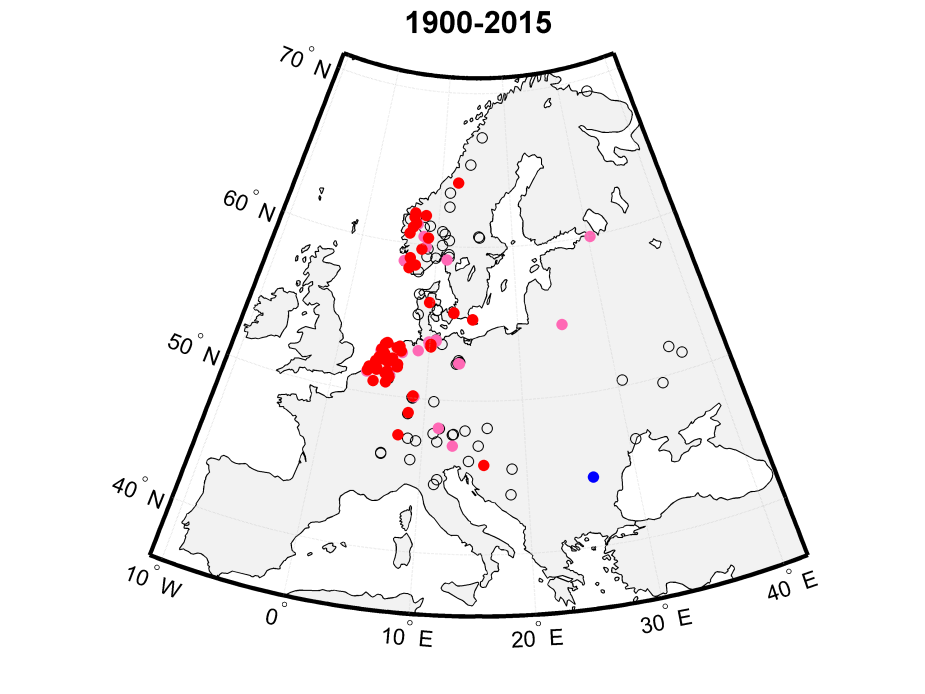


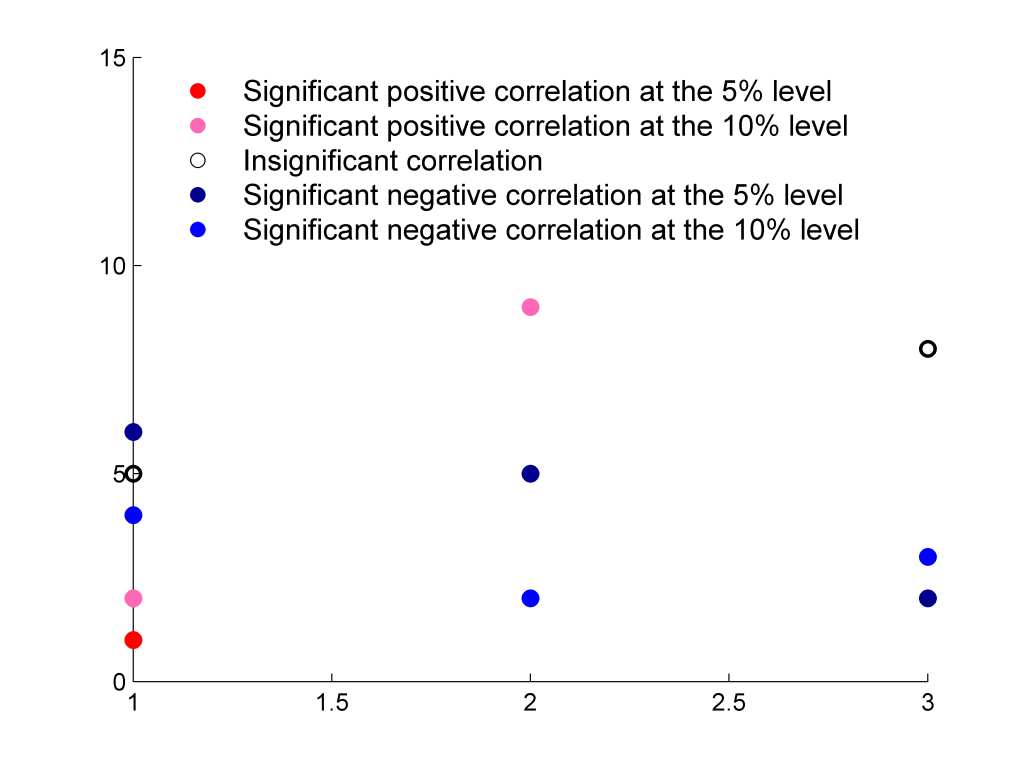

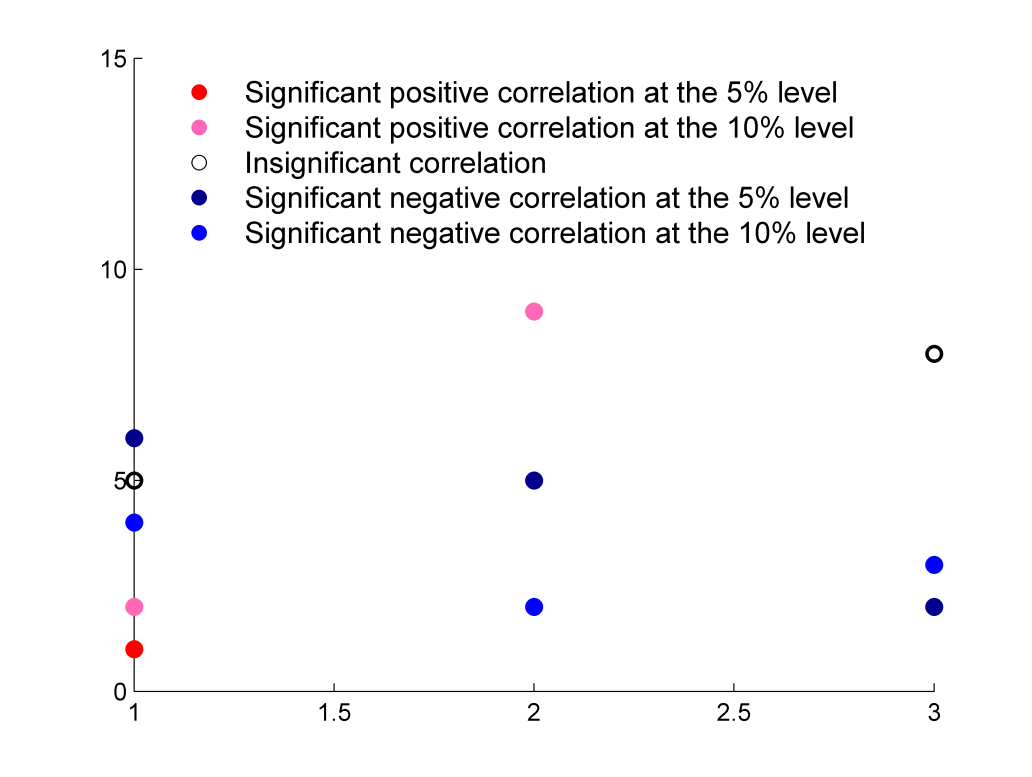


**Figure S6.** Concurrent correlation between the decadal anomalies of daily raingauge precipitation extremes and NAO for the winter season, using long-term records from 263 and 187 stations for the periods 1925-2015 and 1900-2015, respectively. The maps were generated using the software MATLAB (version 2014b) <http://www.mathworks.com/products/matlab/>.


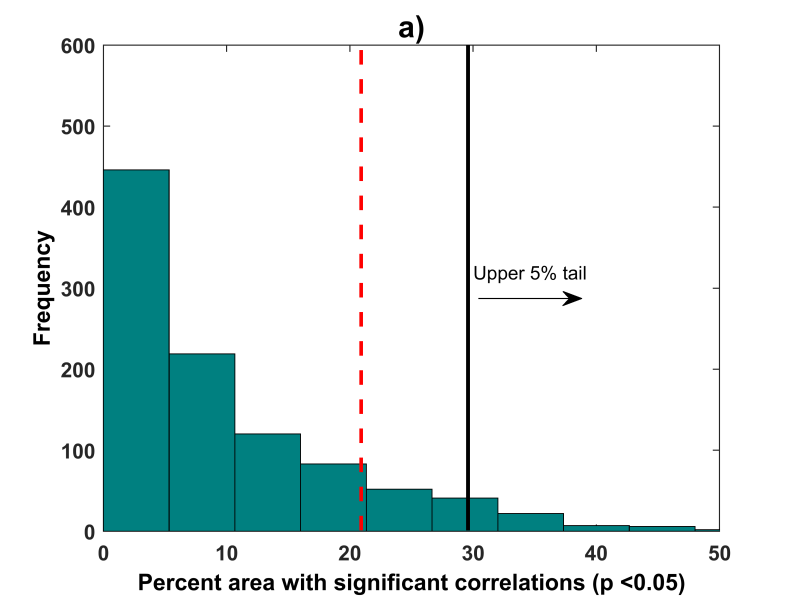

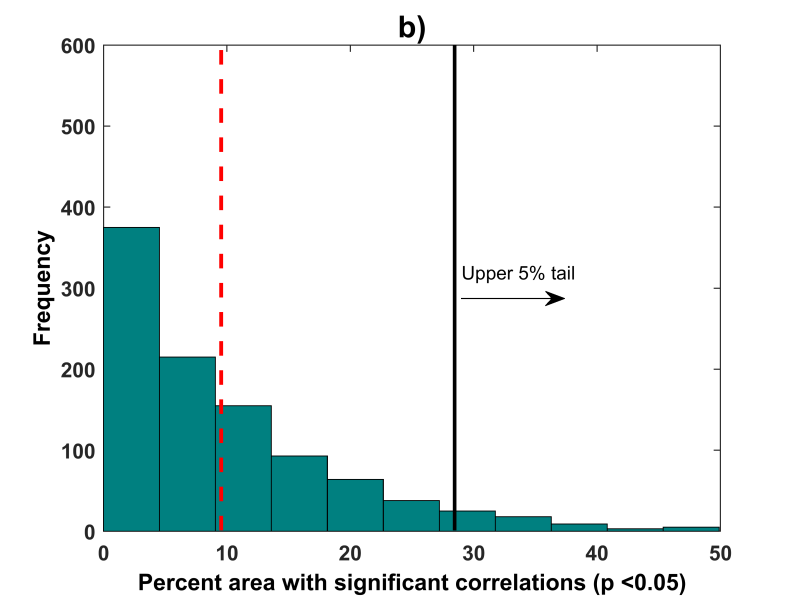


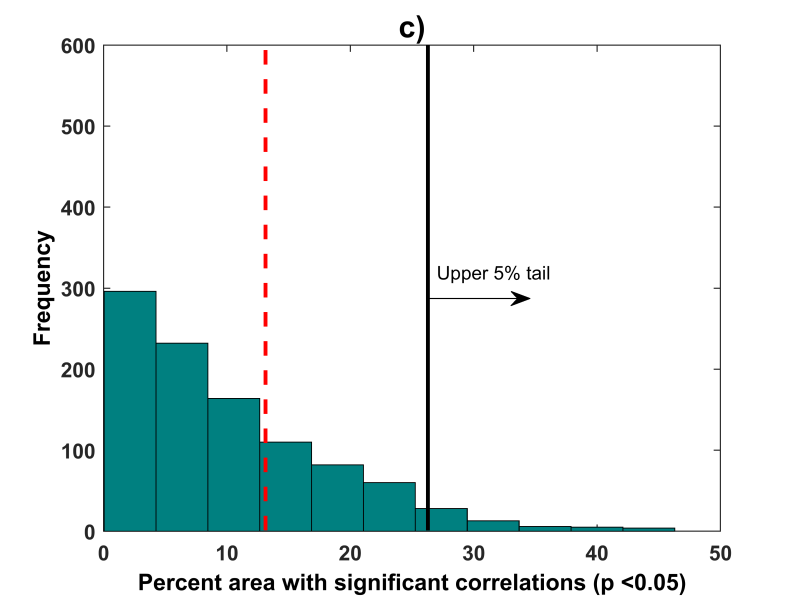


**Figure S7.** Histogram of the percent of European area with significant correlations at the 0.05 level between the anomaly of daily E-OBS precipitation extremes and the randomly resampled anomaly for winter NAO (a), winter SOI (b) and summer WeMO (c). The upper 5% tail of the histogram is indicated by vertical black solid line. The observed percent of area with significant correlation between extreme precipitation and climate indices (Figure 3) is indicated by vertical dashed line (a: winter NAO-winter extreme precipitation; b: winter SOI-winter extreme precipitation; c: summer WeMO-summer extreme precipitation).


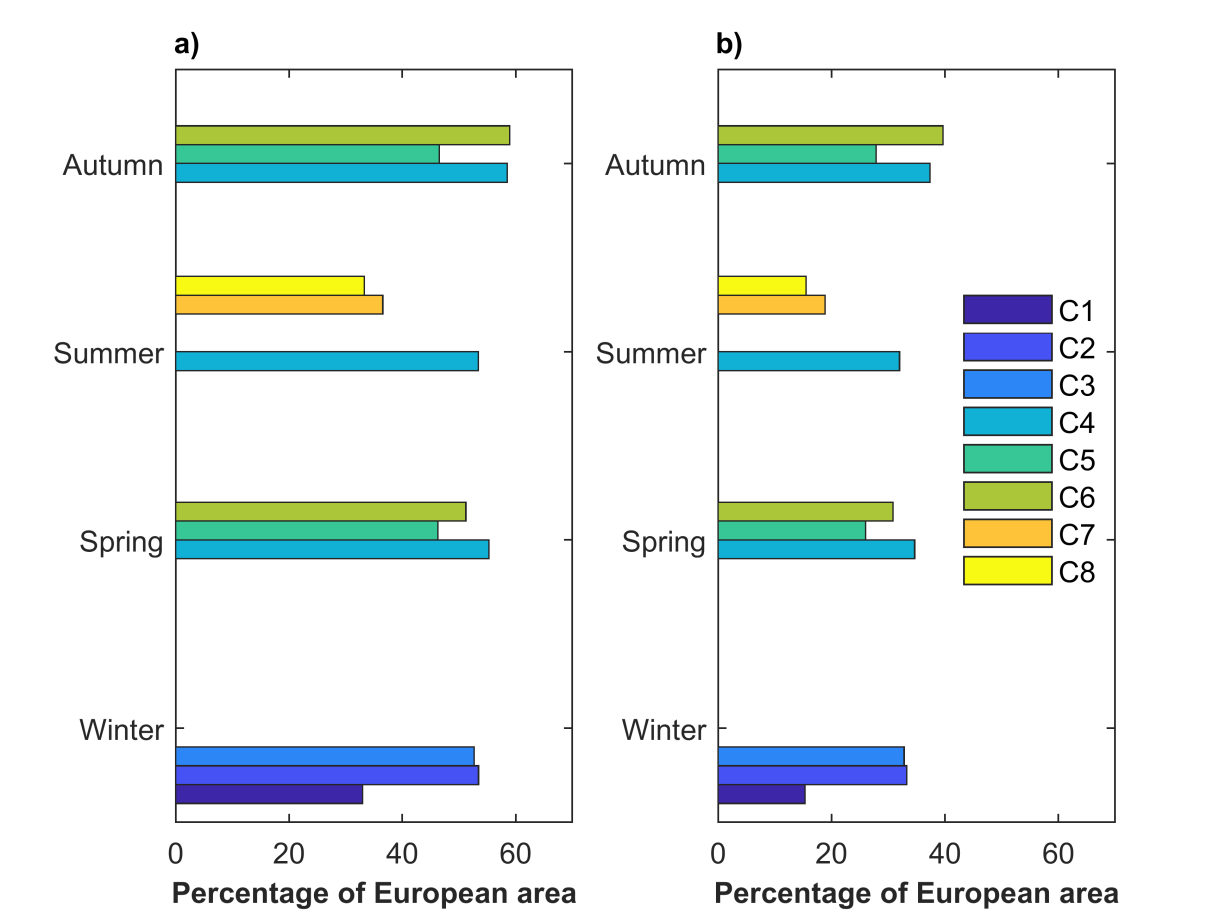


**Figure S8.** Percent of European area with a significant correlation for multivariate analysis at the 90% (a) and 95% (b) confidence levels. In the legend, ‘C’ refers to Combination.

**
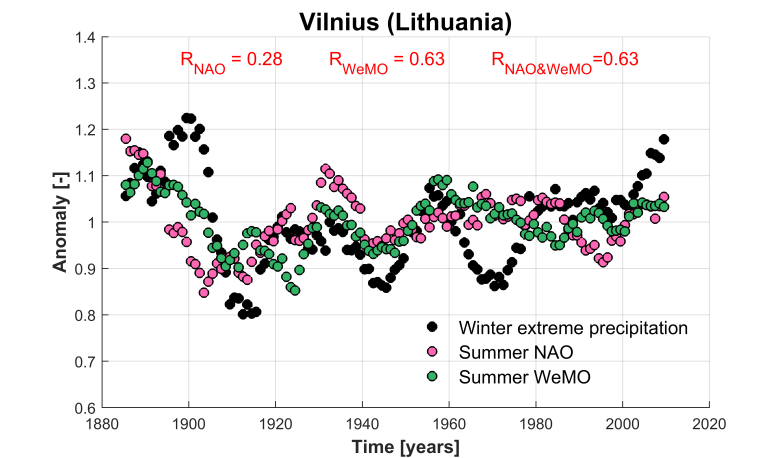

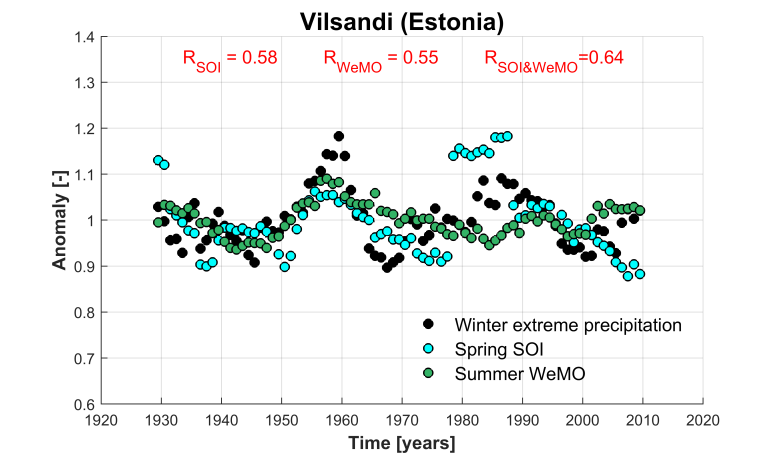

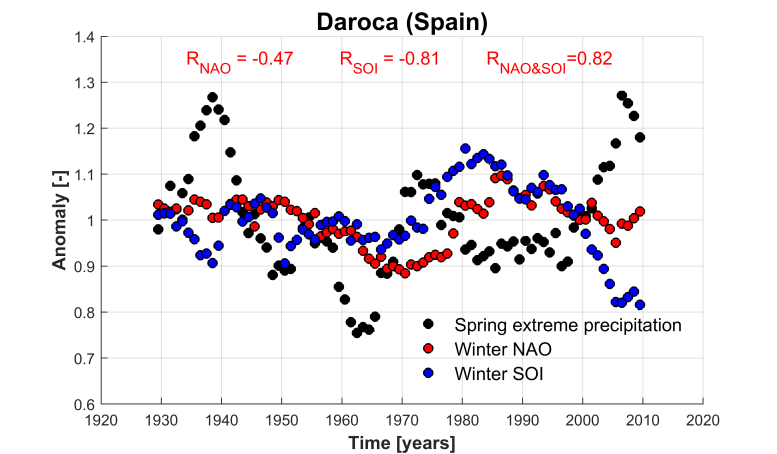

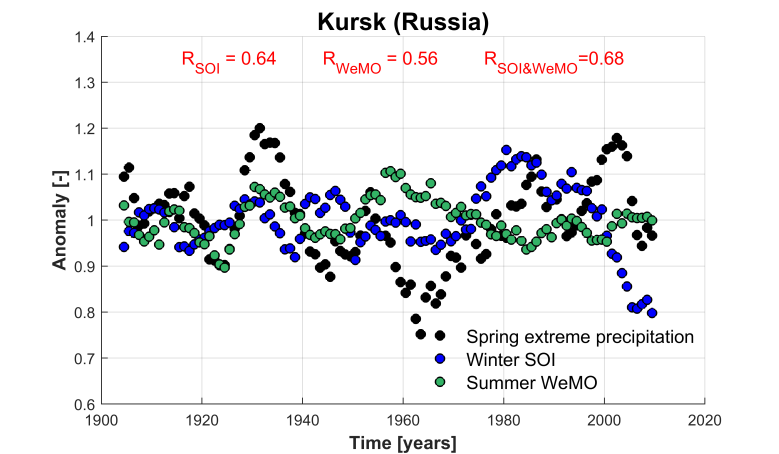

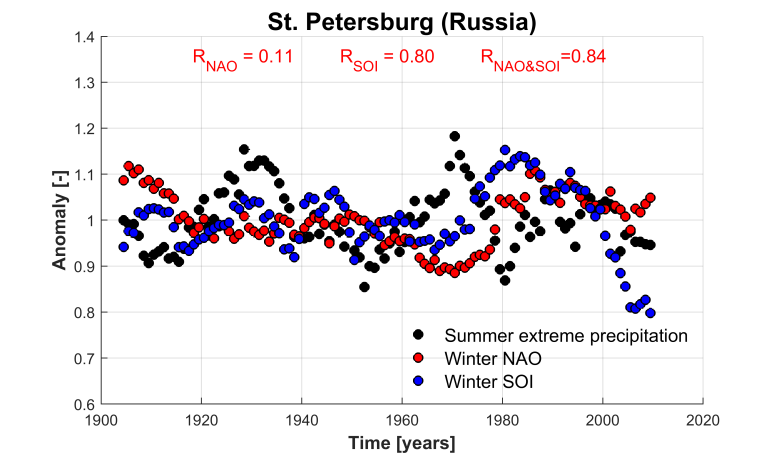

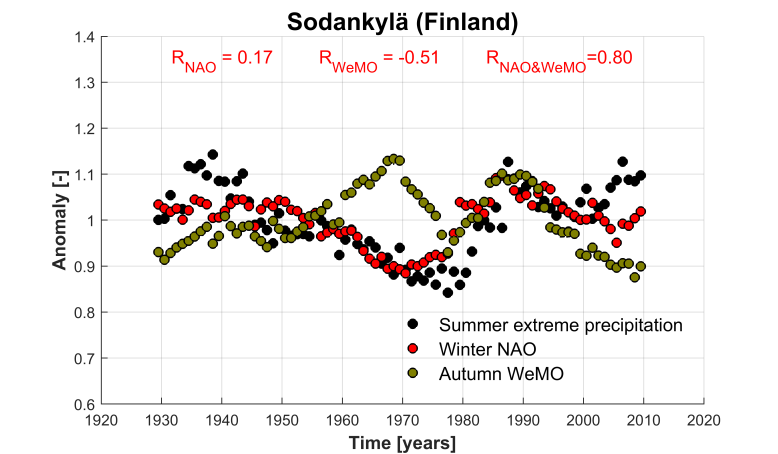

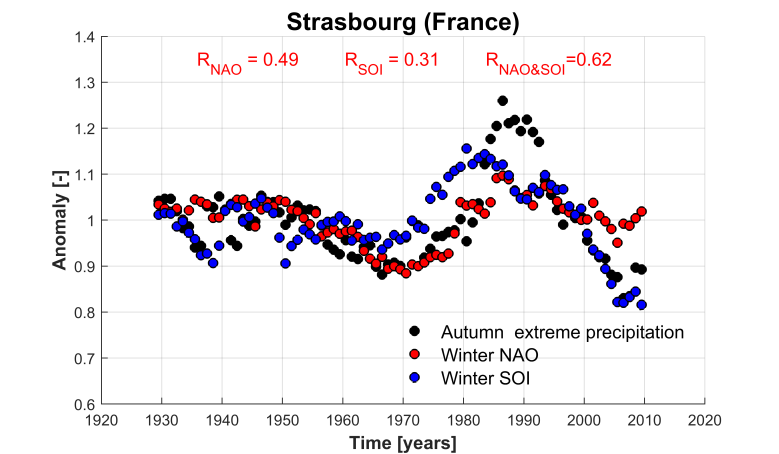

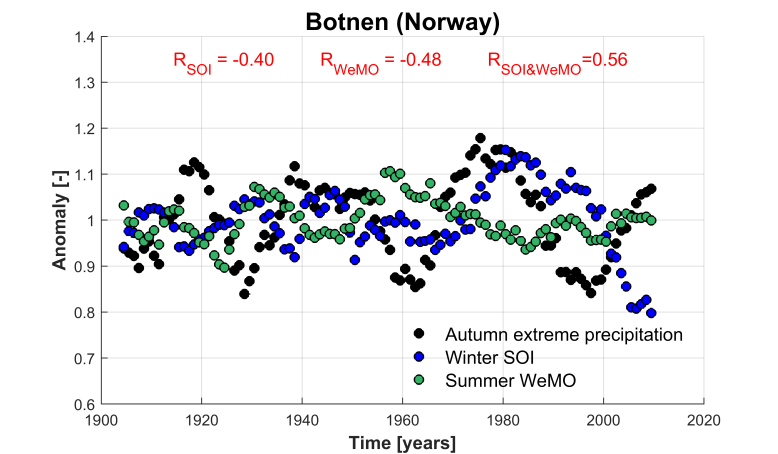
**

**Figure S9**. Relationship between extreme precipitation variability at some selected European stations and climate indices for different seasons. Bivariate and multivariate correlation coefficients are shown with red color on the top of each panel.

**Table S1.** Summary of the used datasets.

| Dataset | Variable | Temporal coverage |
| --- | --- | --- |
| E-OBS | Precipitation | 1950-2015 |
| European raingauges | Precipitation | 1900-2015* |
| NOAA 20th-Century Reanalysis | SLP | 1851-2014 |

* For Figures S4 and S9, precipitation data from some raingauges with longer records were used.

**Table S2.** Validation of E-OBS extreme precipitation anomaly based on raingauge extreme precipitation anomaly for different European regions and seasons using bias (%) and correlation (r) statistics.

| Region^1^ | Country | Location^2^ | Winter | | Spring | | Summer | | Autumn | |
| --- | --- | --- | --- | --- | --- | --- | --- | --- | --- | --- |
|  |  |  | r | Bias | r | Bias | r | Bias | r | Bias |
| Northern Europe | Finland | Sodankyla | 0.94 | 5.2 | 0.98 | 4.9 | 0.94 | 3.1 | 0.91 | 3.2 |
|  | Norway | Sogndal | 0.96 | 3.8 | 0.94 | 4.1 | 0.94 | 2.1 | 0.94 | 3.4 |
|  | Sweden | Falun | 0.84 | 2.4 | 0.81 | 4.1 | 0.79 | 2.6 | 0.83 | 5.0 |
|  | Denmark | Copenhagen | 0.82 | 7.4 | 0.89 | 4.2 | 0.90 | 4.3 | 0.74 | 4.1 |
|  | Lithuania | Vilnius | 0.92 | 4.5 | 0.83 | 4.9 | 0.91 | 3.5 | 0.58 | 6.0 |
| Western Europe | Germany | Hamburg | 0.97 | 3.3 | 0.95 | 3.6 | 0.74 | 3.4 | 0.90 | 2.2 |
|  | Belgium | Uccle | 0.94 | 4.3 | 0.74 | 6.4 | 0.83 | 10.5 | 0.70 | 9.8 |
|  | Germany | Frankfurt | 0.92 | 2.4 | 0.91 | 2.8 | 0.95 | 4.1 | 0.95 | 2.4 |
|  | France | Strasbourg | 0.74 | 4.9 | 0.75 | 5.6 | 0.66 | 5.1 | 0.80 | 5.6 |
|  | Austria | Vienna | 0.65 | 5.0 | 0.95 | 4.6 | 0.97 | 3.4 | 0.69 | 4.5 |
|  | Austria | Salzburg | 0.90 | 4.3 | 0.93 | 2.2 | 0.44 | 4.3 | 0.80 | 4.7 |
|  | Switzerland | Basel | 0.72 | 5.1 | 0.82 | 5.0 | 0.55 | 4.9 | 0.61 | 5.7 |
|  | Switzerland | Zurich | 0.79 | 5.8 | 0.94 | 3.0 | 0.94 | 1.9 | 0.57 | 7.4 |
| Eastern Europe | Ukraine | Kiev | 0.95 | 4.2 | 0.60 | 5.6 | 0.85 | 6.5 | 0.84 | 5.4 |
|  | Ukraine | Shepetivka | 0.85 | 7.9 | 0.95 | 7.5 | 0.91 | 2.7 | 0.45 | 9.2 |
|  | Ukraine | Lugansk | 0.93 | 6.9 | 0.74 | 5.5 | 0.80 | 5.9 | 0.97 | 4.9 |
|  | Romania | Bucharest | 0.78 | 4.2 | 0.91 | 4.3 | 0.75 | 3.9 | 0.88 | 11.2 |
| Southern Europe | Slovenia | Ljubljana | 0.85 | 3.2 | 0.75 | 3.0 | 0.44 | 4.9 | 0.63 | 5.7 |
|  | Croatia | Zagreb | 0.92 | 4.5 | 0.68 | 4.1 | 0.64 | 6.0 | 0.82 | 4.0 |
|  | Bosnia and Herzegovina | Sarajevo | 0.72 | 4.2 | 0.78 | 3.1 | 0.58 | 4.5 | 0.60 | 5.4 |
|  | Spain | Zamora | 0.88 | 6.3 | 0.90 | 4.5 | 0.77 | 13.8 | 0.94 | 5.7 |
|  | Spain | Madrid | 0.73 | 6.6 | 0.85 | 6.9 | 0.63 | 15.6 | 0.42 | 5.6 |

^1^Europe is divided into four sub-regions based on the United Nations geoscheme; ^2^The locations are arranged in latitudinal order.

**Table S3.** Different combinations of climate indices and respective variance inflation factor (VIF). The same combinations are used for spring and autumn extreme precipitation anomalies. Combination 4 is used for spring, autumn and summer extreme precipitation.

| Extreme precipitation anomaly | Combination number | Climate index inputs | VIF |
| --- | --- | --- | --- |
| Winter | 1 | Summer NAO & Spring SOI | 1.14 |
|  | 2 | Summer NAO & Summer WeMO | 1.34 |
|  | 3 | Spring SOI & Summer WeMO | 1.00 |
| Spring & autumn | 4 | Winter NAO & Winter SOI | 1.04 |
|  | 5 | Winter NAO & Summer WeMO | 1.83 |
|  | 6 | Winter SOI & Summer WeMO | 1.02 |
| Summer | 4 | Winter NAO & Winter SOI | 1.04 |
|  | 7 | Winter NAO & Autumn WeMO | 1.04 |
|  | 8 | Winter SOI & Autumn WeMO | 1.01 |

**Movie S1.** Decadal anomalies in daily E-OBS extreme precipitation in winter and summer. Red and green colors denote positive and negative anomaly, respectively. The maps were generated using the software ArcGIS (version 10) <http://www.esri.com/products>.

Supplementary References

1. DeLong, K. L., Quinn, T. M., Taylor, F. W., Lin, K. & Shen, C. C. Sea surface temperature variability in the southwest tropical Pacific since AD 1649. *Nat. Clim. Change* **2**, 799-804 (2012).
2. Li, J. *et al.* El Niño modulations over the past seven centuries. *Nat. Clim. Change* **3**, 822-826 (2013).
3. Torrence, C. & Compo, G. P. A practical guide to wavelet analysis. *Bull. Am. Meteorol. Soc.* **79**, 61–78 (1998).
4. Grinsted, A., Moore, J. C. & Jevrejeva, S. Application of the cross wavelet transform and wavelet coherence to geophysical time series. *Nonlinear Proc. Geoph.* **11**, 561–566 (2004).
5. Torrence, C. & Webster, P. Interdecadal Changes in the ESNO-Monsoon System. *J. Clim.* **12**, 2679–2690 (1999).
